# Genome-wide association analysis of grain iron and zinc in rice grown under agroclimatic sites with contrasting soil iron status

**DOI:** 10.3389/fpls.2025.1501878

**Published:** 2025-07-07

**Authors:** Sonu, Amit Kumar, Vikram Jeet Singh, Prolay Kumar Bhowmick, Shekharappa Nandakumar, Sunaina Yadav, Subbaiyan Gopala Krishnan, Ranjith Kumar Ellur, Haritha Bollinedi, Ashok Kumar Singh, Kunnummal Kurungara Vinod

**Affiliations:** ^1^ Division of Genetics, ICAR-Indian Agricultural Research Institute, New Delhi, India; ^2^ Division of Crop Sciences, ICAR Research Complex for North Eastern Hill (NEH) Region, Meghalaya, Umiam, India; ^3^ Department of Seed Science and Technology, Acharya Narendra Deva University of Agriculture and Technology, Ayodhya, India

**Keywords:** grain iron, iron toxicity, biofortification, GWAS, haplotype analysis

## Abstract

**Introduction:**

Iron (Fe) content in soil can influence rice cultivation, inciting responses ranging from deficiency to toxicity. Fe toxicity is a major constraint, particularly in areas where acidic soils predominate. Grain Fe content along with Zn is a major contemporary breeding objective in rice in order to tackle micronutrient deficiency. There is no information available on the influence of soil Fe levels, normal and excess, can influence grain micronutrient contents, particularly in rice genotypes that are tolerant to excess soil Fe.

**Methods:**

In this study, a subset of 170 rice germplasm lines from the 3K panel were evaluated for grain Fe and Zn concentrations in brown rice across three different locations. Additionally, the response of these lines to Fe toxicity was assessed at one location.

**Results:**

Significant phenotypic variation for both traits was observed. Fe toxicity led to increased grain Fe content but decreased Fe uptake efficiency (IAE), suggesting an adaptive mechanism to limit excess Fe absorption in the rhizosphere. Five significant single-nucleotide polymorphisms (SNPs) associated with grain Fe (*qGFe1.1* ^ADT^, *qGFe2.1* ^BPN-S^, *qGFe8.1* ^ADT^, *qGFe12.1* ^ADT^, and *qGFe12.2* ^BPN-N^) were identified on chromosomes 1, 2, 8, and 12, while one SNP associated with grain Zn (*qGZn12.1* ^BPN-N^) was detected on chromosome 12. These SNPs co-localized with major genes and QTLs involved in heavy-metal homeostasis and transport, including *OsMT2D* and *Os12g0435000*. Superior haplotypes for two candidate genes were identified, with the analysis revealing their frequencies and allelic effects in different subgroups. Two marker-trait associations (MTAs), *qGFe12.1* ^ADT^ and *qGZn12.1* ^BPN-N^, were validated in an F_2:3_ population using linked SSR markers.

**Discussion:**

These validated MTAs provide valuable genetic resources for biofortification breeding programs aimed at increasing Fe and Zn concentrations in rice grains, addressing micronutrient deficiencies among rice-dependent populations.

## 1 Introduction

Rice serves as a staple food source for approximately 3.5 billion people across the globe, contributing to 23% of the per capita global energy intake and supplying 16% of dietary protein (Hoogenkamp et al., 2017). However, the increasing global population is putting tremendous demand on rice production worldwide under escalating challenges of shifting climate. Globally, abiotic stresses such as drought, salinity, submergence, nutritional deficiency, and heavy metal toxicity threaten food security for billions of rice-dependent people. As a primary impediment to rice productivity, the stresses singly or in combination demand the need for novel stress-tolerant rice cultivars (Sandhu et al., 2019; Kaewcheenchai et al., 2021; Theerawitaya et al., 2022).

Heavy metal ions, particularly excess iron (Fe), aluminum (Al), and cadmium (Cd), severely affects plant growth, development, and yield. Over 40% of the world’s arable land is affected by acidic soils, particularly in Asia, sub-Saharan Africa, and South America, which are primary rice-growing regions. These acidic soils often contain toxic levels of Fe and Al, impairing root system development, disrupting nutrient uptake, and causing oxidative damage to plant cells. Due to the negative impacts of climate change, 18% of the total rice-cultivable land on a global scale has come under Fe toxicity problem. Fe toxicity can reduce yields by up to 50% in severely affected areas. Ionic stresses limit growth and grain yield by competing with essential nutrient elements, especially interfering with phosphorus (P) uptake.

In India, which contributes to ~24% of the global rice production, ecosystem diversity is a major challenge in boosting rice productivity (Prasad et al., 2017). Some of the large rice production zones in India such as eastern India are plagued with several stress factors, such as drought, high temperature, low light, soil acidity, salinity, nutrient deficiencies, and toxicities (Kumar et al., 2017). Soil acidity is a major problem in Northeast Indian soils, where excess Fe and Al impede rice production. Fe-containing acid soils develop due to podsolization by leaching of sesquioxides in high rainfall (Patra et al., 2022). The Fe content is significantly impacted by various soil factors, such as texture, pH, electrical conductivity (EC), and organic matter content (Zeng et al., 2011). Soil pH significantly impacts the solubility and accessibility of Fe (Schmidt et al., 2019). In India, approximately 11.7 million hectares of rice-growing areas are affected by excess Fe, including Assam (2.43 million hectares), Kerala (Kuttanad region), and Odisha, where yield losses range from 10% to 100% depending on severity. In Assam, rice yield stands at 2.17 tonnes per hectare, while Kerala faces challenges due to waterlogging and extreme soil acidity. Odisha’s lowland rice fields suffer significant productivity declines due to Fe toxic soil, potentially interfering with crop production (Sonu et al., 2024; Ebimol et al., 2023; Regon et al., 2021; Reddy et al., 2019). Similar conditions are observed in other rice-growing regions worldwide. In Africa, hundreds of hectares of land are being abandoned due to Fe toxicity. A substantial portion of rice cultivation areas in countries such as China, the Philippines, Thailand, Malaysia, and Indonesia are also threatened by Fe toxicity (Ullah et al., 2023).

Understanding the effect of different soil Fe status on grain micronutrient accumulation, especially of Fe, is an interesting aspect of micronutrient homeostasis in rice plants. Since rice grains accumulate Fe sourced from the soil, the paradoxical situation of low or excess soil Fe is likely to affect the Fe content in the grain. Despite significant progress in identifying the genetic mechanisms that regulate Fe uptake, transport, and grain accumulation in rice, there is limited information on the genetic potential and control of grain Fe assimilation in rice when grown in soils with varying Fe levels. It is also interesting to know the effect of defense strategies to excess Fe on micronutrient uptake and assimilation as well as of the genomic regions regulating those responses. This knowledge can guide the development of rice varieties that are both resilient to abiotic stresses and capable of accumulating optimal levels of essential micronutrients, thereby addressing productivity and nutritional challenges. This is particularly important in the context of increasing global Fe toxicity in rice-growing soils, while rice remains as an important vehicle for supplementing essential micronutrients like Fe and Zn. Furthermore, most previous studies on Fe toxicity tolerance in rice have primarily based on artificial screening under controlled environments, leaving a gap in information under natural field conditions. In this study, we evaluated a diverse set of rice germplasm under different Fe conditions and characterized grain Fe and Zn assimilation vis-à-vis their response to Fe toxicity. We have also evaluated the genetic regulation of the responses’ traits using genome-wide association studies (GWAS).

## 2 Materials and methods

### 2.1 Plant materials and experimental setup

The study utilized 174 genotypes, comprising a subset of the 3K genome panel (Wang et al., 2018) of 170 genotypes and four check varieties. Initiated with 192 genotypes (188 germplasm lines and four checks), the study could continue only with 174 genotypes, as 14 genotypes suffered establishment issues rendering them unusable in the downstream analyses. The checks were contrasting excess Fe-responsive genotypes, Shahsarang (tolerant), Megha SA2 (tolerant), Pusa 44 (sensitive), and IR64 (sensitive). Shahsarang is a medium-tall (100–110 cm), non-lodging, and medium-duration (120–130 days) indica rice variety with moderate resistance to blast disease and high tolerance to Fe toxicity. Shahsarang performs well in acidic and lowland soils, and its grains are notable for their high Fe and Zn content. Megha SA2, another variety tolerant to excess Fe (Sonu et al., 2024), is a late-maturing cultivar well suited to mid- to low-altitude lowland ecosystems. It produces medium-aromatic, long slender grains with awns and red-colored kernels. Among the sensitive checks, IR64 is an early-maturing, high-yielding variety with comparatively lower grain micronutrient levels, while Pusa 44 is a long-duration, semi-dwarf, high-yielding cultivar known for its sturdy stem. The subset assembly had predominantly Indian origin genotypes comprising cultivars, landraces, and breeding lines (**Supplementary Table S1**). All materials were sourced from the ICAR-Indian Agricultural Research Institute (IARI), New Delhi.

Field evaluations were conducted at three diverse agro-ecological locations in India. These sites were New Delhi (DEL), Aduthurai (ADT), and Barapani (BPN) having four sites with varying soil Fe levels. At DEL, the experiment was conducted during *Kharif* 2022–2023 at the experimental plots of the Division of Genetics, ICAR-IARI (28°38′ N; 77°10′ E; 246 m), while at ADT, the field at the IARI Rice Breeding and Genetics Research Centre (RBGRC, Tamil Nadu) was used (11°00′ N; 79°28′ E; 41 m) in Rabi 2022–2023. Located at the experimental farm of the ICAR-Research Complex for North Eastern Region, Umaim, Meghalaya (25°41′ N; 91°54′ E; 980 m), at BPN, there were two different lowland field conditions. Site 1 was with normal Fe (24.6 ppm) (BPN-N) and site 2 was with excess Fe-stressed (72.0 ppm) (BPN-S) conditions. DEL soil was relatively low in Fe (16 ppm) (Rani et al., 2021) (site 3), while ADT had moderate soil Fe content (24.6 ppm) (site 4) (Jeyasingh et al., 2023). The soil Zn content at all of the sites was moderate, with DEL having a DTPA-extractable Zn content of 2.5 ppm, BPN with 1.96 ppm, and ADT with 1.2 ppm. The soil types and pH varied across sites, with DEL having sandy loam soil (pH 8), ADT with alluvial soil (pH 6.28), and BPN having red loam acid soil (pH 5.63 for BPN-N and pH 5.2 for BPN-S).

The field experiments have been laid out in augmented randomized complete block design at all three sites under irrigated transplanted conditions. The nursery was raised on elevated beds for 21 days and transplanted into well-puddled field with a spacing of 20 × 15 cm. The field was divided into four blocks, with 47 genotypes in each block to accommodate the initial panel of 188 genotypes. The check genotypes were replicated four times over four blocks. Each entry was transplanted in 0.9-m^2^ plots of three rows having 10 plants each. The entire trial was managed with recommended agronomic practices at all of the locations. Experimental layout and randomization were made using PBTools v1.4 (Sales et al., 2013). As previously mentioned, only 174 genotypes were used further for analyses.

A QTL validation population was developed by crossing Shahsarang/IR64. These two parents were selected based on their contrasting responses to Fe toxicity rather than being among the top 12 high-iron and zinc accessions identified in the study. Cross was made in 2020, and F_1_ seeds were grown and tested using SSR markers to confirm hybridity. 192F_2_ plants were raised along with the parental lines at ICAR-IARI, New Delhi, during the *Kharif* season of 2021–2022. The plants were selfed and harvested separately. The F_2:3_ seeds were evaluated in the lowland area at ICAR-NEH region, Umiam, Meghalaya, during *Kharif* 2022–2023, and phenotyping was done for grain Fe and Zn.

### 2.2 Analysis of grain Fe and Zn content

At maturity, the grains were harvested from each genotype separately from five uniform-looking plants per plot from each of the four experiments. The harvested grains were pooled per genotype, cleaned by removing any discolored or ill-filled spikelets and sun-dried for 3 days to bring to uniform moisture content. The grains were then dehusked using Satake^®^ THU35C-T testing husker. Grain Fe (GFe) and Zn (GZn) levels were directly determined using Hitachi^®^ X-Supreme 8000 (Oxford Instruments Plc., UK) energy-dispersive X-ray fluorescence (ED-XRF) spectrometer calibrated as per Paltridge et al. (2012) for high-throughput screening of brown rice (Chandu et al., 2020). To this, 5 g of dehusked and cleaned rice kernels was placed in 30-mm aluminum sample cups and sealed with Poly-4 XRF film. The samples were uniformly distributed by gentle shaking before analysis. The GFe and GZn contents were recorded in mg/kg.

### 2.3 Soil Fe estimation

Soil Fe content (SFe) was estimated using the diethylenetriamine pentaacetate (DTPA) method (Lindsay and Norvell, 1978). Soil samples were collected from the experimental plots and dried sufficiently. Moreover, 20 g of air-dried sample was thoroughly mixed with 40 mL of DTPA solution, allowed to stand for 1 to 2 h, and centrifuged. The supernatant was extracted and filtered with Whatman 41 filter paper, and the filtrate was analyzed for Fe concentration using atomic absorption spectrophotometry.

### 2.4 Grain iron assimilation efficiency

For the purpose of understanding the influence of soil Fe content on grain Fe concentration, we propose an index, grain iron assimilation efficiency (GIAE), following similar indices calculated for other nutrients (Xu et al., 2012; Saito et al., 2021).


$$\text{GIAE}=\frac{\text{GFe content}}{\text{Total Fe in Soil}}\times 100$$

### 2.5 Statistical analysis

Site-wise analysis of variance (ANOVA) was used to compute coefficients of variation for phenotypic (PCV) and genotypic (GCV) components and broad sense heritability (*H* ^2^). R package *augmentedRCBD* (Aravind et al., 2023) was used in the computations. Using the pooled data, best linear unbiased predictors (BLUPs) were generated using a restricted maximum likelihood (REML) approach using the *lme4* package integrated with the software PBTools v1.4 (IRRI 2014). Jitter box plots were drawn in R using *ggplot2* package. Correlation analysis was done and presented graphically in R using *corrplot* package. Graphical analysis of genotype (G), site (E), and genotype-by-site interaction (GSI) was done using GGE biplot analysis using *GGEbiplotGUI* package and factorial regression analysis (FRA) was done using GEA-R software (https://data.cimmyt.org). FRA particularly emphasized how different co-factors of different environments influence the genotype performance (Denis, 1988; Van Eeuwijk et al., 1996; Vargas et al., 1999). A factorial regression (FR) model that incorporates environmental covariates into the genotype × environment interaction (GEI) can be expressed as:


$${\text{Y}}_{ij}=\text{µ}+{\text{g}}_{i}+{\text{e}}_{j}+\sum_{g=1}^{H}{\text{Z}}_{ih}{\text{ζ}}_{jh}+{\text{ϵ}}_{ij}$$

where Y*_ij_* is the response variable of the *i* ^th^ genotype (*i*=1,…,*I*) in the *j* ^th^ environment (*j*=1,…,*J*). µ represents the grand mean, g*_i_* and e*_j_* are the genotype and environment deviations respectively from the grand mean; Z*_ih_* are the environmental covariates; ζ*_jh_* are the genotype factor; *H* (*H*< *J*) is the number of environmental covariates, and ϵ*_ij_* is the error term.

### 2.6 Population structure and linkage disequilibrium

To estimate the population structure of the test germplasm, genome-wide data of 3,341,271 SNPs were downloaded from the Rice-SNP Seek database (Alexandrov et al., 2015). Filtered across taxa for a maximum of 10% missing call rate and across the sites for minor allele frequency of 0.05 and heterozygosity >0.3, a total of 140,487 markers were used for analysis. Chromosome-wise SNP distribution was visualized using an SNP density plot in SRplot software (Tang et al., 2023). The number of subgroups in the association mapping panel was estimated using the LEA-R package (Frichot et al., 2014) along with vcfR and PCA. Analysis was performed with assumed subgroups (K) ranging from 1 to 10, replicated 10 times. The total number of ancestral populations was determined using cross-entropy and the elbow method (Bholowalia and Kumar, 2014). PCA was conducted with GAPIT (Lipka et al., 2012), and the significant number of PCs was determined using a scree plot. Pairwise LD was calculated using a 50-bp sliding window in TASSEL 5.0, and LD (*r*²) was plotted against marker distance (Bradbury et al., 2007). Only *r*² values with *p <*0.05 were considered for LD decay analysis, and the LD decay plot was constructed in R 4.2.3 as per Remington et al. (2001).

### 2.7 GWAS analysis

Bayesian information and linkage disequilibrium iteratively nested keyway (BLINK) model adopted in GAPIT package was used to identify marker–trait associations (MTAs) because of its high computing efficiency and statistical power. Significant MTAs were identified after a Bonferroni multiple test correction calculated from the reciprocal of the total number of markers used for analysis [*p* < 4.78E-07; -log10(*p*) > 6.32]. Circular Manhattan plots and symphysic Q-Q plots were used for the graphical presentation of significant MTAs. The percentage of phenotypic variance explained (PVE) by individual SNP was calculated through the single-marker analysis. These MTAs were named following QTL naming conventions.

### 2.8 Assessment of the novelty of identified MTAs

The marker interval which harbors significant marker–trait association across the locations was studied further to identify putative candidate genes for grain Fe and Zn content. The probable expressed genes present between the marker positions were downloaded from the Rice Annotation Project Database (RAP-DB) and compared as to the physical positions with those of the previously reported quantitative trait loci (QTLs). In addition to the literature survey, the Gramene QTL database (https://archive.gramene.org/qtl/) and KnetMiner (Hassani-Pak et al., 2021; https://knetminer.com) were searched to identify the physical locations of the previously reported QTLs.

### 2.9 Validation of identified quantitative trait loci

The parental genotypes of the validation population were tested for parental polymorphism for the SNP-linked SSR markers using microsatellite (SSR) markers, the segregation ratio of polymorphic markers was checked using chi-square test, and distorted markers were removed (Zhang et al., 2010). Phenotypic data was tested for ANOVA, and best linear unbiased predictors (BLUPs) were generated through the REML approach using the *lme4* package integrated with the software PBTools v1.4 (Sales et al., 2013) with genotypes as random variables in the model. The F_2_ genotypic data was regressed on the phenotypic BLUPs of F_2:3_ and the corresponding individual F_2_s to perform single-marker analysis. The markers which showed significant variation among the parental classes for the target trait were considered validated for the corresponding linked QTL.

## 3 Results

### 3.1 Phenotypic variation for grain micronutrients

**Figure 1:**
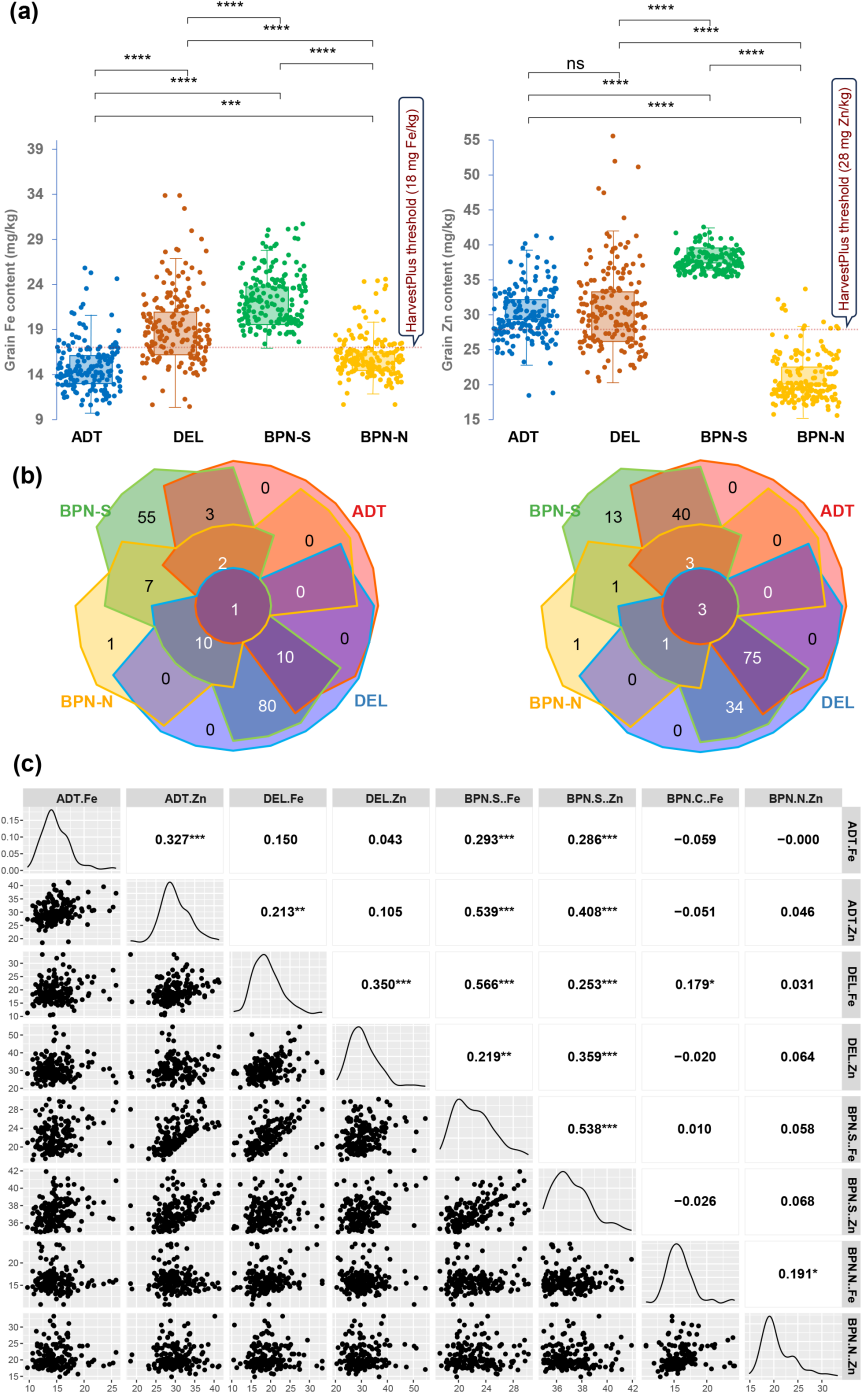
**(a)** Boxplots showing the distribution of grain Fe and grain Zn content under four sites, **(b)** Venn diagram showing common genotypes that show the average grain nutrient content above HarvestPlus threshold values, and **(c)** correlogram of grain Fe and Zn content across four sites having varying soil Fe contents. The sites are coded as DEL for Delhi, ADT for Aduthurai, and BPN for Barapani.

Pooled analysis of variance showed significant variations for genotype (G), site (S), and GSI components for GFe and GZn (**Table 1**). Variation due to checks and genotype at BPN-N was not significant for GFe, while grain GZn exhibited significant variation. Boxplots across all of the sites showed the distribution for both traits, confirming the quantitative inheritance of the trait (**Figure 1A**). The average GFe and GZn content across the sites is presented in **Supplementary Table S2**. GFe showed the highest mean of 22.3 mg/kg at BPN-S, with a range of 17.3 to 30.2 mg/kg, followed by DEL with an average of 17.9 mg/kg and a range of 10.5 to 33.3 mg/kg. No significant difference was observed for GFe content at ADT and BPN-N with average GFe of 14.9 and 15.9 mg/kg, respectively. Similarly, GZn content with the highest average was observed at BPN-S condition with a value of 37.2 mg/kg and a range from 34.9 to 41.9 mg/kg, followed by DEL, ADT, and BPN-N. Genetic variability analysis revealed that the highest PCV and GCV for GFe were observed to be highest at ADT (22.0 and 20.8, respectively), followed by DEL (19.6 and 18.1), BPN-S (19.2 and 17.1), and BPN-N (15.6 and 13.0). GZn displayed the highest PCV and GCV at BPN-N (19.0 and 18.3, respectively), followed by BPN- S (16.4 and 16.3), DEL (16.2 and 15.3), and ADT (14.6 and 14.0). Highest broad sense heritability was observed at ADT for GFe, while the lowest was observed for BPN-N, and the GZn maximum heritability was observed at BPN-S and the lowest at DEL (**Table 1**).

**Table 1:** Analysis of variance for the grain micronutrient content under four sites with varying levels of soil Fe content.

Source	GFe				GZn			
	ADT	BPN-N	BPN-S	DEL	ADT	BPN-N	BPN-S	DEL
Treatment	7.6*	5.4	8.9*	18.6**	23.8**	15.9**	5.8**	41.9**
Check	8.3*	6.6	48.1**	58.9**	31.9**	77.2**	7.3*	64.9**
Test	7.5*	5.3	8.3*	15.7**	14.2**	11.5**	2.17	33.2**
Test vs. check	22.6**	6.1	43.2**	427.5**	1618.2**	654.5**	613.8**	1476.8**
Block	0.1	3.4	6.6	5.1	3.9	2.1	4.9*	5.4
Residuals	1.16	1.6	2.67	2.1	1.17	0.9	0.68	2.4
Genotype (G)	2.7***				1.8**			
Site (S)	10.3**				42.5**			
GxS	4.6***				13.4**			
Residual	2.5				1.45			
Mean ± SE (ppm)	14.9 ± 0.2	15.9 ± 0.2	22.3 ± 0.3	17.9 ± 0.3	29.8 ± 0.3	21 ± 0.3	37.2 ± 0.4	30.1 ± 0.4
Minimum	9.7	10.7	17.3	10.5	18.5	14.9	34.9	20.5
Maximum	25.8	24.3	30.2	33.3	41.3	33.3	41.9	54.6
CV%	7.1	7.5	8.6	7.78	4.1	5.1	2.2	5.4
SD	3.3	2.5	4.6	3.6	4.8	4	6.0	5.0
GCV%	20.8	13.0	17.1	18.1	14.0	18.3	16.3	15.3
	(high)	(medium)	(medium)	(medium)	(medium)	(medium)	(medium)	(medium)
PCV%	22.0	15.6	19.2	19.6	14.6	19	16.4	16.2
	(high)	(medium)	(medium)	(medium)	(medium)	(medium)	(medium)	(medium)
*h* ^2^(BS)	89.6	76.7	80.0	84.5	92.1	92.4	98.2	89.3
	(high)	(high)	(high)	(high)	(high)	(high)	(high)	(high)
GAM	40.6	3.9	31.6	34.2	27.7	7.6	33.2	30.0
	(high)	(high)	(high)	(high)	(high)	(high)	(high)	(high)

GFe, grain Fe content in ppm; GZn, grain Zn content in ppm; ADT, Aduthurai; BPN-N, Barapani- normal; BPN-S, Barapani-stressed; DEL, Delhi; CV, coeﬃcient of variation; SD, standard deviation; GCV, genotypic coeﬃcient of variation; PCV, phenotypic coeﬃcient of variation; *h2*(BS), heritability in broad sense; GAM, genetic advance over mean. *, **, and *** indicate significant variation at p < 0.05, p < 0.01, and p < 0.001 levels, respectively.

According to the critical grain micronutrient thresholds for biofortification set by HarvestPlus—grain Fe >18 mg/kg and grain Zn >28 mg/kg—several genotypes were found to exceed these levels when grown at different sites, particularly at sites BPN-S and DEL. For grain Zn, in addition, numerous genotypes exceeded the critical level at ADT also. However, the genotypes did not consistently maintain high micronutrient levels across all sites. For grain Fe, only one genotype, IRG173, consistently showed over the threshold GFe across all sites. In the case of GZn, three genotypes—IRG189, IRG192, and IRG72—consistently exceeded the threshold across all locations (**Figure 1B**).

### 3.2 Interrelations grain micronutrient contents across sites

Pearson’s correlation coefficient between the grain micronutrient contents showed a significant association between sites (**Figure 1C**). The GFe and GZn contents between ADT and BPN-S showed a significant positive association. The GFe content at DEL showed a positive trend with that of other sites as well as with the GZn content except for BPN-N. Except for the positive association between GFe and GZn at BPN-N, GFe at this site did not show any relation with the micronutrient status at other sites, whereas the GFe recorded at BPN-S has had a significant association with GZn content at ADT, DEL, and BPN-N. Other than these, the GZn content at different sites showed a low association with the grain micronutrient contents of other sites. Exception on this were the associations observed between GZn contents BPN-S and those of ADT and DEL. Interestingly, no associations in the grain micronutrient contents could be observed between normal and stressed conditions at BPN, signifying the effect of Fe toxicity in grain micronutrient accumulation.

### 3.3 Fe assimilation efficiency

**Figure 2:**
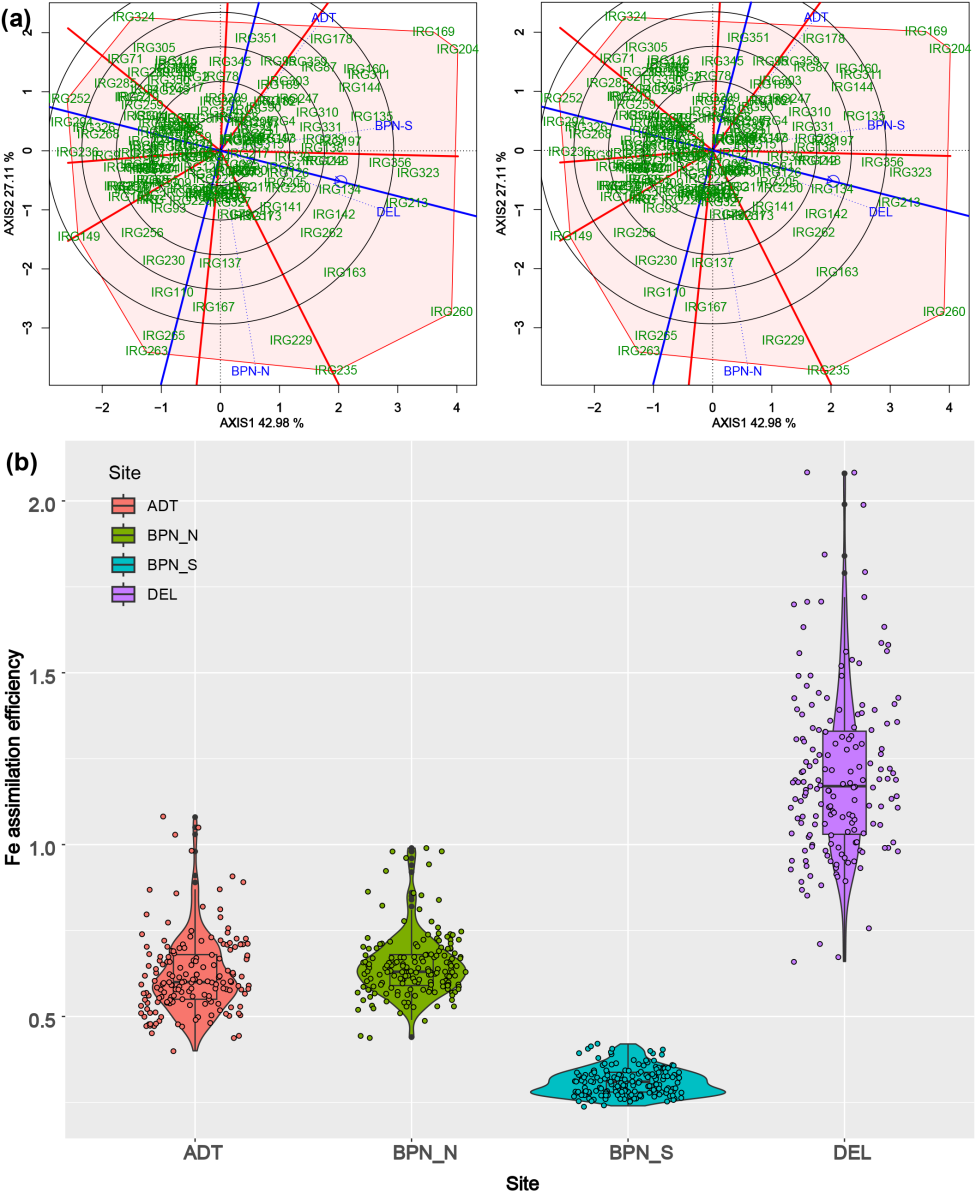
**(a)** Combined GGE biplots describing the genotype and environment dispersion for grain micronutrient content at four sites with varying soil Fe levels. **(b)** Violin plots showing the distribution of Fe assimilation efficiency among the panel genotypes under sites with varying soil Fe levels.

The average Fe assimilation efficiency (IAE) of genotypes varied significantly between the sites, and so were the variances. IAE was highest in DEL (1.20) and lowest at BPN-S (0.31). The IAE at ADT (0.62) and BPN-N (0.65) conditions were close and were intermediate to that at the other two sites. The sample variance also varied between 0.04 (BPN-S) and 0.25 (DEL) (**Figure 2B**).

### 3.4 Site soil characters and genotype-by-environment interactions

Site-wise soil analysis revealed that the DEL site had a pH of 8.0, EC of 1.2 dS/m, and 16 mg/kg soil Fe content. ADT soil was alluvial, having a pH of 6.3, EC of 1.7 dS/m, and 24.0 mg/kg Fe content. The BPN-S site was highly acidic with a pH of 4.5–5.2, having a Fe content of 72.0 mg/kg and EC of 0.11 dS/m. The BPN-N site soil had a Fe content of 24.6 mg/kg with a pH range of 5 to 6 and EC 0.18 dS/m (BPN-N). The FRA demonstrated the varied levels of significance in the influences of G, S, and GSI components on both GFe and GZn. Partitioning GSI further, all three soil variables, pH, EC, and soil Fe were found to have a significant influence (**Table 2**). Interaction of genotype × soil pH had a profound share of 47.9% in genotype × site interaction for GFe content, followed by SFe (36.1%) and EC (32.4%). A similar pattern of influence was observed on GZn also, in which genotype × soil pH interaction had 58.2% share in the GSI, followed by EC (30.9%) and SFe (21.8%).

**Table 2:** ANOVA for factorial regression showing component variances for the fitted models, describing the genotype × site effects of soil factors in determining grain Fe and Zn content.

Parameters	GFe				GZn			
	Variance	Pr(>F)	%GSI	AIC	Variance	Pr(>F)	%GSI	AIC
Site (S)	100.6	0.0	–	–	652.6	0.0	–	–
Genotype (G)	7,633.8	0.0	–	–	10,190.7	0.0	–	–
Site × rep	0.3	0.9	–	–	1.0	0.4	–	–
G × S	11,015.2	0.0	–	–	20,895.9	0.0	–	–
G × pH	5,274.6	0.0	47.9	–	12,171.0	0.0	58.2	–
G × SFe	3,975.4	0.0	36.1	–	4,547.1	0.0	21.8	–
G × EC	3,570.2	0.0	32.4	–	6,457.2	0.0	30.9	–
Residuals	153.1	–	–	–	152.4	–	–	–

GFe, grain Fe content in ppm; GZn, grain Zn content in ppm; EC, electrical conductivity (dS/m); SFe, soil Fe content in ppm; AIC, Akaike information criterion; %GSI, the proportion of genotype by site interaction explained; Pr(>F), F-probability.

The biplots of GGE analysis indicated the differential response of the test genotypes under different sites. In both cases, DEL was the closest to the average environment axis (AEA), and BPN-N remained the farthest. Among the genotypes, IRG169 and IRG204 showed high GFe content under BPN-S, while IRG260 showed higher GFe under DEL location. There was a distinct difference among the sites for Fe content, while there was no significant variation among the sites for GZn content. IRG193, IRG247, and IRG197 were the top rankers in GZn but were found unstable across environments. However, genotypes such as IRG142, IRG205, IRG163, IRG207, etc., were found to be stable as well as having higher GZn. In the case of GFe, IRG218 and IRG134 were found stable (**Figure 2A**).

### 3.5 Linkage disequilibrium and population structure

**Figure 3:**
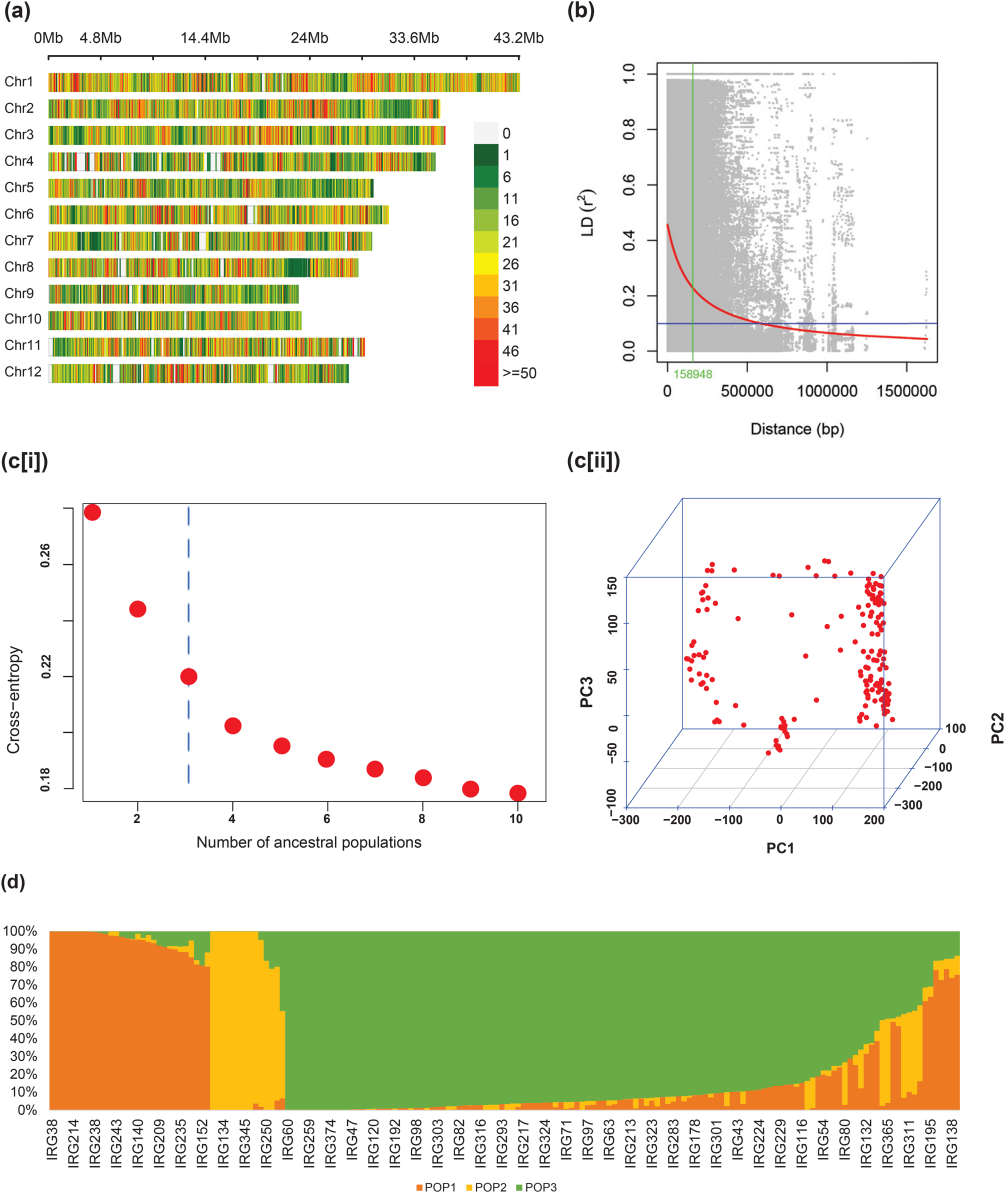
**(a)** Genome-wide distribution of SNPs in the association panel, **(b)** LD decay and the cutoff LD, **(c)** number of ancestral populations by (i) elbow and (ii) PCA 3D, and **(d)** bar plot showing population admixing using the co-ancestry coefficients.

The chromosome-wise distribution of 140,487 SNPs among the panel of 170 genotypes revealed that the markers were distributed across with an average distance of 2.66 kb (**Table 3**). The highest marker density was found on chromosome 1 with an average marker distance of 1.99 kb and the lowest was on chromosome 9 with an average marker distance of 3.73 kb (**Figure 3A**). Linkage disequilibrium (LD) calculated based on the *r* ^2^ values denoted a critical LD of 158.9 kb based on the 95th percentile of the cumulative pairwise distances between markers on each linkage group. The markers present between a physical distance of less than 159 kb tend to inherit together as a single haplotype unit (**Figure 3B**). The cross entropy from the estimated admixture coefficients plotted against the expected number of subpopulations revealed a significant fall in the slope at *K* = 3, revealing the elbow point (**Figure 3C**). The three sub-populations were designated as POP1, POP2, and POP3. A PCA analysis performed on the genotypic data also indicated a significant presence of three groups in the population structure, explaining about 40% of the total variation (**Figure 3D**). With a maximum cutoff value of 0.95 for admixture (co-ancestry) coefficients (Q), 49.4% of admixtures were identified in the whole population. When plotted in the bar chart, POP1 had 30 genotypes, of which 18 genotypes were with less than 5% admixing, and the remaining 12 had 5% to 41% admixing. POP2 contained 14 genotypes, nine of which were least admixed and the remaining five were admixed. Among 126 genotypes in POP3, 59 were least admixed and 67 were admixtures (**Figure 3E**). The details of the genotypes in the three subpopulations are provided in **Supplementary Table S3**.

**Table 3:** Chromosome-wise distribution of SNPs used in genome-wide analysis.

Chromosome	No. of markers	Length (Mb)	Average marker distance (kb)	Marker density per Mb
1	21,978	43.25	1.99	508.2
2	14,529	35.94	2.47	404.3
3	13,842	36.41	2.63	380.2
4	12,057	35.50	2.94	339.6
5	9,417	29.90	3.18	314.9
6	13,877	31.24	2.25	444.2
7	10,898	29.97	2.75	363.6
8	10,075	28.44	2.82	354.3
9	6,151	22.94	3.73	268.1
10	8,840	23.21	2.63	380.9
11	9,053	29.02	3.21	312.0
12	9,770	27.53	2.82	354.9
Total	140,487	373.35	2.66	376.3

Mb, million base pairs; kb, kilobase pairs.

### 3.6 MTAs for grain micronutrient content

**Figure 4:**
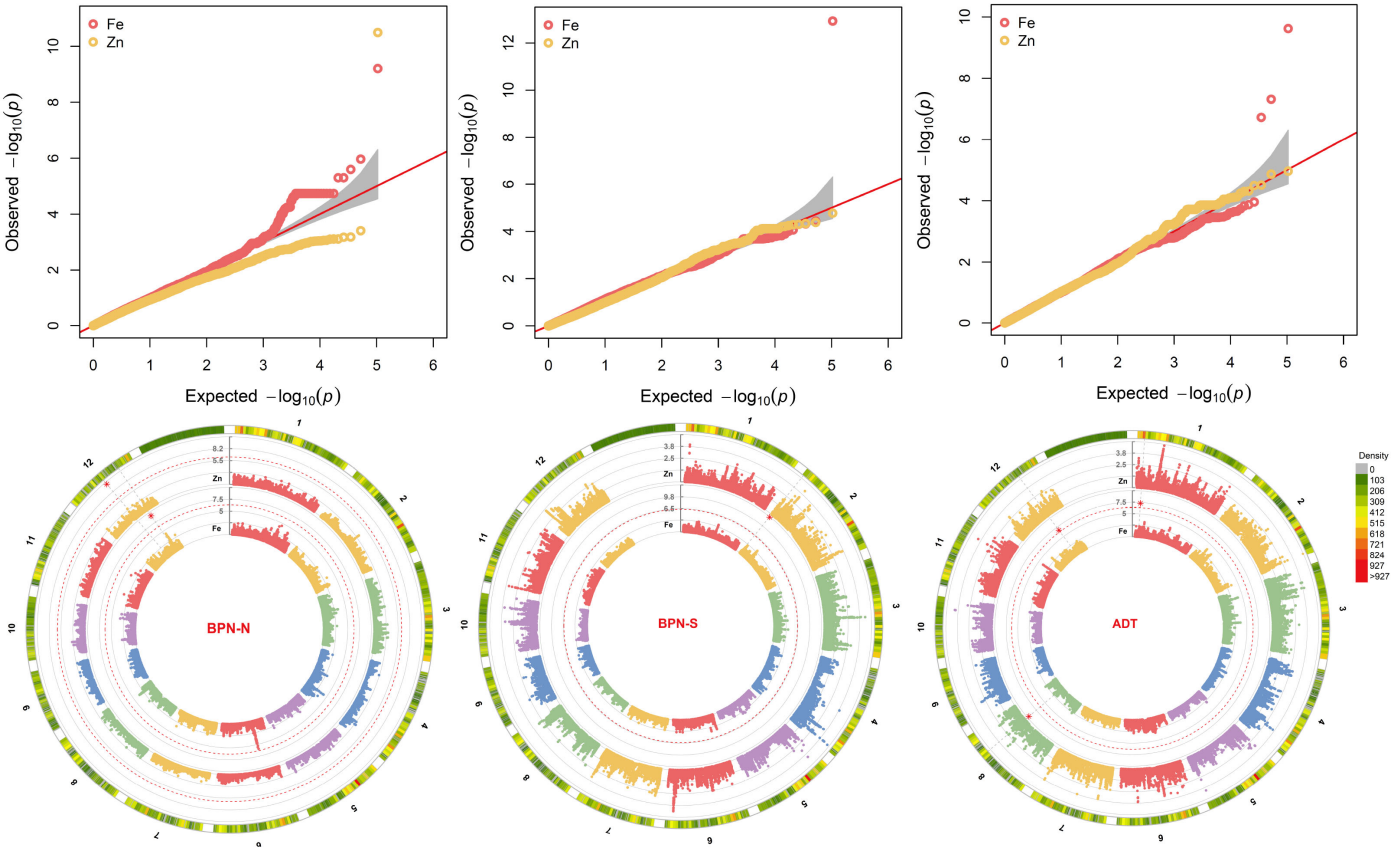
Circular Manhattan and Q-Q plots for the marker–trait associations (MTAs) identified for grain micronutrients in multisite evaluation under varying soil Fe concentrations. The traits are coded as GFe for grain Fe content in ppm and GZn for grain Zn in ppm, and the sites are coded as ADT for Aduthurai, DEL for Delhi, and BPN for Barapani. S indicates a plot with Fe toxicity, and N is for a plot with normal Fe level.

The Q-Q plots for all the traits showed the expected distribution of log_10_(*p*) that implied perfect control of false discovery (**Figure 4**). Among the six MTAs identified, five were mapped for GFe and one for GZn content. Three MTAs detected for GFe at ADT were distributed on chromosomes 1(*qGFe1.1* ^ADT^), 8 (*qGFe8.1* ^ADT^), and 12 (*qGFe12.1* ^ADT^) and positioned respectively at 2.6, 19.4, and 18.5 Mbp. These MTAs explained a phenotypic variance of 10.8%, 8.3%, and 9.7%, respectively. At BPN -S, the only MTA detected was on chromosome 2 (*qGFe2.1* ^BPN-S^) located at 0.48 Mbp, which explained a PVE of 42.3% for GFe. There were two MTAs identified under BPN-N both located on chromosome 12. One of these MTAs was associated with GFe (*qFe12.2* ^BPN-N^) and was located at the physical position of 21.2 Mbp, explaining 41.7% variation. The other MTA was associated with GZn (*qGZn12.1* ^BPN-N^) and was located at 14.2 Mbp, accounting for 32.2% of the total phenotypic variance (**Table 4**).

**Table 4:** Marker-trait associations (MTAs) identified for grain micronutrient content under multi-site evaluation.

Location	MTA	Trait	Chrom	Position	*p*-value	Effect	-log_10_(*p*)	PVE%
ADT	*qGFe1.1* ^ADT^	GFe	1	2693943	4.80E-08	0.39	7.32	10.84
	*qGFe8.1* ^ADT^	GFe	8	19370641	2.40E-10	0.33	9.62	8.27
	*qGFe12.1* ^ADT^	GFe	12	18503677	1.88E-07	0.2	6.7	9.74
BPN-S	*qGFe2.1* ^BPN-S^	GFe	2	488238	1.18E-13	0.28	12.9	42.27
BPN-N	*qGFe12.2* ^BPN-N^	GFe	12	21260572	6.24E-10	0.33	9.21	32.23
	*qGZn12.1* ^BPN-N^	GZn	12	14185774	3.21E-11	0.21	10.5	41.71

GFe, grain Fe content in mg/kg; GZn, grain Zn in mg/kg; ADT, Aduthurai; DEL, Delhi and BPN, Barapani; S indicates plot with Fe toxicity; N is with normal Fe level.

### 3.7 *In silico* search for candidate genes

A total of 92 annotated gene models were identified within the haplotype surrounding the identified MTAs. These included 42 candidate genes belonging to different protein family classes such as auxin transporters, cation efflux proteins, heavy-metal-associated domains, nicotinamine synthase, 6-phosphoribosyl transferase domains, iron-regulated metal transporters, and zinc ion binding proteins. *qGFe1.1* ^ADT^ was found to be associated with genes like *OsMT2D* (*Os01g0149200*) and *OSMT2A* (*Os01g0149800*) encoding metallothionein (MT) and controlling iron and zinc homeostasis. *qGFe2.1* ^BPN-S^ was found to be associated with Cytochrome p450, CYP1, and HPL1 gene encoding heme-binding protein, while *qGFe8.1* ^ADT^ was found proximal to genes *OsHMP39*, *HMP39*, *OsHIPP46*, *OsaHIP46*, *HIP46*, *OsHMP40*, *HMP40*, *OsHPP7*, *OsaHPP07*, and *HPP07* (*Os08g0403300*) encoding heavy-metal-associated protein 39 and heavy-metal-associated isoprenylated plant protein 46. Candidate genes around *qGFe12.1* ^ADT^ included genes encoding *OSM* protein (oxidative stress management genes), while candidate genes around *qGFe12.2* ^BPN-N^ were in close proximity with the genes *OSGRX28* and *OSGRX29* and genes encoding metal binding protein. The MTA was also found to be associated with genes such as *VIL1* (*Os12g0533500*) and *VIP5* (*Os12g0535900*) associated with grain yield and biomass. The genes associated with *qGZn12.1* ^BPN-N^ include *OsFUSED*, *CCD1*, and *LAP1*, controlling traits like pollen development, lipid transport, and zinc ion binding protein (**Supplementary Table S4**).

### 3.8 QTL validation

**Figure 5:**
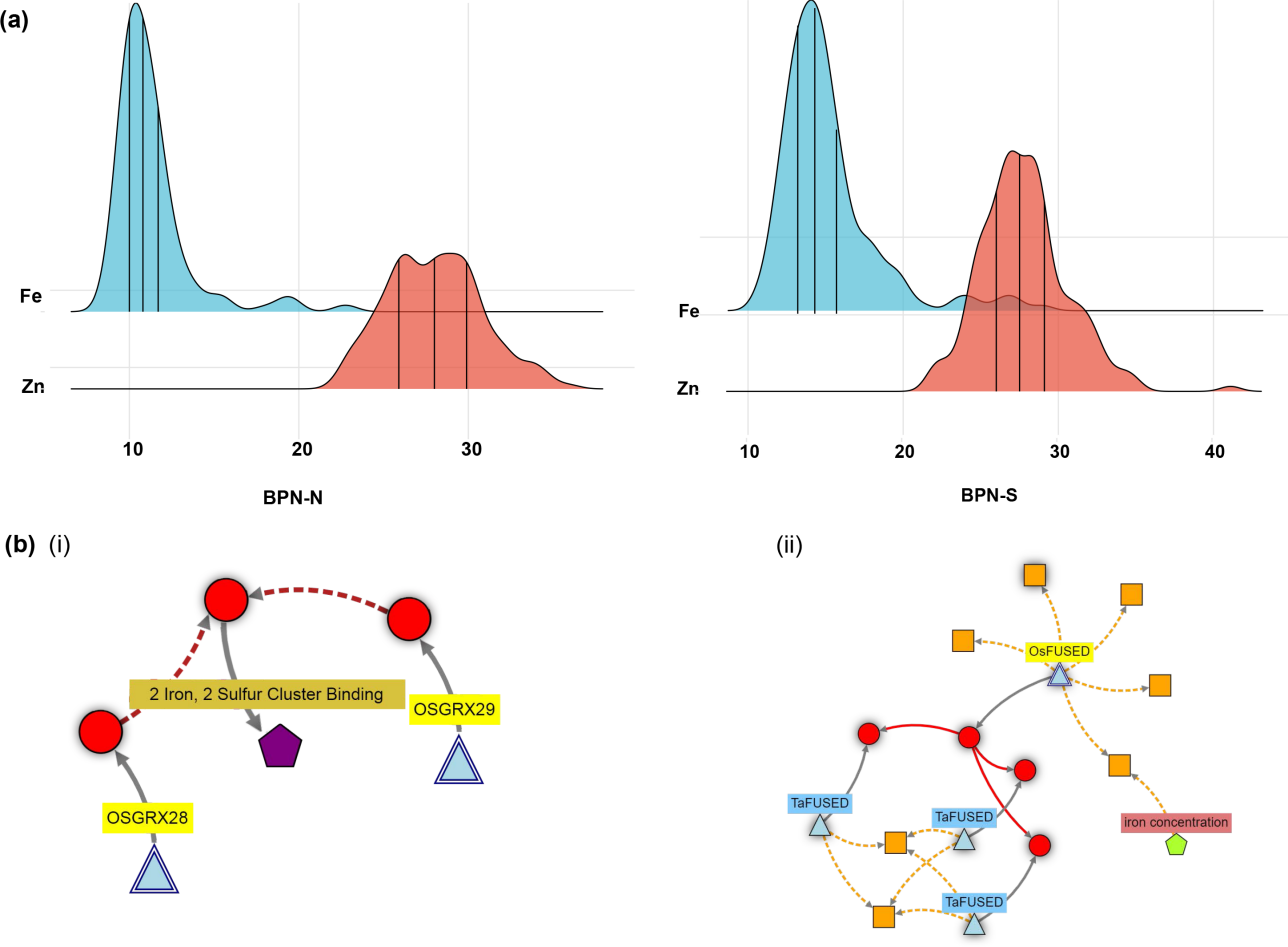
**(a)** Gradient ridges showing the density distribution of Fe response traits among the F2:3 population of the cross, Shahsarang/IR64 under two levels of soil Fe at Barapani. **(b)** Network knowledge graphs showing the gene networks associated with validated MTAs for traits (i) grain Fe content and (ii) grain Zn content.

The ANOVA of the F_2:3_ population derived from Shahsarang/IR64 showed significant differences for GFe and GZn contents among the segregating progenies. The frequency distribution histogram of F_2:3_ demonstrated that both of the traits were normally distributed in the population, confirming the quantitative inheritance pattern (**Figure 5A**). The phenotypic evaluation revealed GFe ranging from 8.1 to 31.1 mg/kg, with a mean value of 15.2 mg/kg and high PCV and GCV, while GZn ranged from 22.1 to 39.0 mg/kg, with a mean value of 28.0 mg/kg and low GCV and medium PCV. Both of the traits exhibited high heritability (**Supplementary Table S6**).

Using SSR markers, two QTLs out of six, *qFe12.2* ^BPN-N^ and *qZn12.1* ^BPN-N^, were validated. There were 30 SSR markers present in the vicinity of the MTA region on chromosome 12. A polymorphism survey between the two parents identified six polymorphic markers between them (**Supplementary Table S7**). The marker, RM1986, showed a significant association with the GFe under both conditions (BPN-N and BPN-S) and RM179 with GZn at BPN-S. The genotypes which carry the homozygous allele of IR 64 (RM1986) showed an average GFe of 12.5 mg/kg at BPN-S and 10.6 mg/kg at BPN-N, whereas genotypes which carry homozygous Shahsarang alleles showed 17.6 mg/kg at BPN-S and 12.6 mg/kg at BPN-N. The PVE of the 12:21260572 SNP was found to be 55.3% and 16.4% with additive effects of 0.9 and 2.5 mg/kg at BPN-S and BPN-N, respectively, indicating that the positive QTL allele was contributed by Shahsarang. Genotypes with homozygous RM179 alleles of IR 64 showed an average GZn content of 27 mg/kg, whereas genotypes with homozygous Shahsarang alleles showed 31.5 mg/kg. The PVE of the marker was 42.7%, and the additive effect was found to be 2.2 mg/kg, indicating that the QTL was contributed by Shahsarang (**Table 5**).

**Table 5:** Single-marker analysis showing the validation of MTA-linked SSR markers among the segregation population of the cross, Shahsarang/IR64 under different screening systems.

Location	Trait	SNP	Marker	Chrom	MSS	*R* ^2^ (%)	Additive effect
BPN-N	GFe	12:21260572	RM1986	12	881.9**	55.3	2.5
BPN-S	GFe	12:21260572	RM1986	12	75.0**	16.4	0.9
BPN-N	GZn	12:14185774	RM179	12	391.2**	42.7	2.2

The site codes were BPN-N, Barapani-normal, and BPN-S, Barapani-stressed.

GFe, grain Fe content in ppm; GZn, grain Zn content in mg/kg.

### 3.9 Gene network associated with the validated MTAs

The *qFe12.2* ^BPN-N^ was found networked with two thioredoxin fold domain containing protein genes, *Glutaredoxin 28* (*OsGRX28*) and *Glutaredoxin 29* (*OsGRX29*), located within the 21.4–21.5-Mbp region. Another prominent network associated with *qZn12.1* ^BPN-N^ was linked to the *OsFUSED* gene (*Os12g0433500*) (**Figure 5B**).

## 4 Discussion

Among the several nutrients essentially required for rice, two important mineral micronutrients, viz., Fe and Zn, are considered critical to human nutrition. The dietary availability of these minerals is essential to thwart micronutrient malnutrition (Bollinedi et al., 2020). Fe supports energy metabolism by playing a critical role in oxygen transport, while Zn is essential for immune function, cell division, and enzyme activation. Biofortification is the process of enhancing the nutritional value of staple food crops by increasing their content of essential vitamins, minerals, and other beneficial nutrients. This approach aims to improve the nutrient supply in the human diet. Fe and Zn are two key micronutrients commonly targeted in biofortification efforts. These enhancements can be achieved through both biological and artificial methods, though biological approaches are generally considered more sustainable. Promoting agricultural practices that improve soil health is a key strategy to enrich the nutrient content of food grains. In this context, variations in soil Fe levels across different food-producing regions are likely to have a direct impact on the Fe concentration in harvested grains. Soil nutrient availability is dependent on soil, water, and climatic parameters, and crop nutrient assimilation is genetically controlled. Under this complex scenario, it is difficult to draw a direct relation between nutrient status and grain nutrient content. This study therefore bridges the information on how rice genotypes accumulate GFe and GZn under varying soil Fe levels and how their responses are genetically regulated.

The study utilized 174 genotypes adapted to various rice-growing zones of India, particularly, the northeast, where soil and climatic variations are high. The study sites fell under three different agroclimatic zones, with varying levels of Fe availability. The three agroclimatic zones represented in this study included the Eastern Himalayan Region (EHR), where BPN was located; the Trans-Gangetic Plain Region (TGPR), encompassing DEL; and the East Coast Plains and Hills Region (ECPHR), which has ADT. These regions were highly characteristic concerning their ecological diversity and cropping pattern. EHR was hilly with predominantly acidic soils having a sub-humid climate, TGPR had a semiarid climate having an extreme temperature range, and EPHR had an alluvial, loam, and clayey soils with a sub-humid maritime climate. In all of the regions, rice was a traditional crop, but intensity was varying. In EHR, rice cultivation was on terrace systems where shifting cultivation (*Jhum*) was practiced, while TGPR followed the wheat–rice cropping system, accommodating rice during the Kharif season. Rice cropping intensity at the EPCHR region was the highest with up to three crops per year. Due to the significant effect of cultivation practices on the soil fertility status, we used managed fertilization in all of the sites, except for Fe. The combination of genotypes and the sites could therefore bring out an array of genotype responses that were studied closely in the context of grain nutrient accumulation. Site-wise variation in nutrient contents has been reported in several previous studies (Huang et al., 2015; Bollinedi et al., 2020; Cu et al., 2021).

The variation among the Fe content was greater than that of Zn, which apparently could be correlated with the native soil nutrient available at different sites. Despite the variations, a significant positive correlation was observed between both traits at all the locations as reported earlier (Dixit et al., 2019; Bollinedi et al., 2020; Cu et al., 2021; Pasion et al., 2023). The concomitant mobilization of Fe and Zn into the grains has been reported in several crops in the past. Although the exact mechanism remains obscure, there is ample evidence that the metal ion transport in plants takes several common regulators across complex genetic pathways (Connorton et al., 2017). One of the regulators is a transcription factor, *ILR3* (IAA-leucine resistant 3), a basic helix-loop-helix DNA-binding protein (*bHLH*) from *Arabidopsis* involved in Fe homeostasis. Another key player is the Zn-regulated, Fe-regulated transporter-like protein (ZIP) family gene, *Zrt*/*Irt3*, established to be involved in the transport of both Zn and Fe. Notwithstanding, many of the Fe ion transport regulators are also involved in Zn mobilization, resulting in co-regulated variation for these two micronutrients in plants (Diaz et al., 2022).

Significant genotypic and GSI were present in both GFe and GZn content in this study, as described earlier by Pandian et al. (2011) in milled rice and by Naik et al. (2020) in brown rice. The grain ion concentration could change with the corresponding levels of the nutrient reservoir in the soil, expressing a direct impact (Pujar et al., 2020). Therefore, the common regulatory mechanism of metal ion transport and assimilation must keep a balance when there is a contrast in the soil nutrient contents, similar to the case in the current study wherein the Fe levels varied relatively higher than the Zn content (CV ranged between 7.1% and 8.6% for GFe as against 2.2% and 5.4% for GZn across locations). GFe content increased with increasing soil Fe concentration at different sites, with the maximum GFe content recorded at the BPN-S location. Notwithstanding, no significant elevation in GZn content could be observed. However, this was in contrast to IAE recorded at various sites. IAE was the lowest in BPN-S, suggesting that high Fe in the soil is not fully translated to GFe. The extreme levels of Fe induced a toxicity response, resulting in a negative effect on the translocation efficiency. Although very few studies have been conducted on the effects of soil Fe and Zn content on micronutrient accumulation in grain, Bierschenk et al. (2020) observed a moderate, albeit statistically insignificant, rise in grain Fe concentrations, while a notable and statistically significant increase in grain Zn concentrations was evident under chronic Fe toxicity stress. We could also observe increased Zn accumulation under Fe toxicity. Frei et al. (2016) also reported a non-significant increase in grain Fe and Zn with acute and chronic stress treatments. Excess Fe in soil affects, in general, the uptake of all the nutrients because of the eventual root system damage (Li et al., 2016). However, root system damage does occur slowly, allowing the uptake of a large amount of Fe by the plants. This will incite competition for nutrients within the plant system, resulting in Fenton reactions, which, in turn, prevent the mobilization of Fe and Zn ions to the developing grains.

Due to the geographical and climatic diversity of the study sites, the soils exhibited considerable variability in properties such as pH and electrical conductivity (EC), with DEL showing a pH of 8.0 and EC of 1.2 dS/m, ADT with a pH of 6.3 and EC of 1.7 dS/m, BPN-S with a pH ranging from 4.5 to 5.2 and EC of 0.11 dS/m, and BPN-N with a pH of 5–6 and EC of 0.18 dS/m. Soil pH plays a significant role in plant assimilation of Fe and Zn, increasing the availability particularly when it is in the acidic range. Since soil pH plays a profound role in solubilization, acidification can release more bound Fe from the soil. It has been estimated that the solubility of Fe falls by ~1,000 times per unit increase in soil pH (Lindsay and Schwab, 1982; Hochmuth, 2011). Under a lower pH, the uptake by rice plants remarkably increases below the pH of 5.0 (Fageria et al., 2008). Establishing this, the remarkable effect of pH and Fe content in soil augmented by EC could be observed on grain Fe accumulation in the present study. On average, the grain Fe content at the BPN-S site was 50% higher than at ADT, while the relative increase compared to DEL was 16%. The contributory effects of these soil parameters on grain Fe accumulation were earlier reported by Pandian et al. (2011) on rice and Joshi et al. (2010) on wheat. Notably, the combined effect of pH and EC was found to majorly influence the accumulation of Zn across environments.

To keep the target of biofortifying the crop varieties, particularly the staple cereals, the International Food Policy Research Institute (IFPRI) has instituted a global program aimed at ending micronutrient malnutrition worldwide by promoting crops that are enriched with mineral nutrients like Fe, Zn, and vitamin A. Known as HarvestPlus, this program has set minimum targets for identifying biofortified rice varieties. The target for grain Fe content is >18 mg.kg^-1^ and for Zn is >28 mg.kg^-1^ in brown rice (Bouis and Saldman, 2017). As per these standards, we could identify one genotype, Bir Bahadur (IRG173), an *aus*/boro accession from Bihar, India, with above critical values for GFe across all locations. Similarly, three accessions, Jugray (IRG189), K17-9-1-1 (IRG192), and Cauvery (IRG72), were found to possess above critical GZn content, across all locations. Jugray is an indica landrace from Chhattisgarh, while K17-9-1–1 is an indica breeding line. Cauvery is an indica variety from Tamil Nadu (Gangadhara et al., 2024). Having shown consistently high grain Fe and Zn across all of the environments, these genotypes can be used as potential donors in the breeding program. Wide variability in rice accessions, especially involving several landraces, has previously been reported particularly in brown rice (Bollinedi et al., 2020; Anuradha et al., 2012; Maganti et al., 2020), identifying genotypes with high Fe and Zn content in the grains.

We have chosen the test assembly of genotypes considering two advantages: (a) being part of 3K genome assembly, their genome data was available; and (b) there was an ample representation of genotypes of Indian origin in the panel. Indian-origin genotypes were kept on the focus because they possessed the best adaptation potential to Indian environments. Six QTLs could be identified from the GWAS associated with GFe and GZn. Three MTAs (*qGFe12.1* ^BPN-N^, *qGFe12.2* ^BPN-N^, and *qGZn12.1* ^BPN-N^) were found distributed 3 to 4 Mb apart on chromosome 12, suggesting the possibility of genetic regulation of both nutrients on the arm of the chromosome. This situation presents the breeders with an opportunity to select for concurrent enhancement of both GFe and GZn from appropriate donors having positive alleles under a breeding program. Interestingly, five of the six MTAs identified were found to be in the vicinity of previously reported QTL regions and candidate genes, while one was novel. MTA identified on chromosome 1 *qGFe1.1* ^ADT^ at 2.7 Mbp was found in very close proximity to *qFe1.1* (RM294A–RM12276) as reported by Swamy et al. (2021). This locus was proximal to *OsPOT* (LOC_Os01g65110.1) coding for proton-dependent oligopeptide transporters that were identified to play a role in the vascular translocation of Fe^2+^–NA complexes (Dixit et al., 2019). *OsMT2a* (Os01g0149800; 2169bp), a metallothionein-like protein type 2 known for their metal-binding and stress–response functions, is also found very close to this QTL (Zhou et al., 2006). MTA identified on chromosome 2, *qGFe2.1* ^BPN-S^, was found near (66 kb) to a candidate gene hydroperoxide lyase 1 (*HPL1*, Os02g0110200) and linked to (0.675 kb) Cytochrome P 89 (*CYP89*, Os02g0108800) gene encoding, heme-binding protein/Fe-ion-binding protein. This MTA showed the highest PVE under Fe toxic plots at BPN-S. MTA on chromosome 8, *qGFe8.1* ^ADT^, was found proximal to heavy metal transport and detoxification genes associated with heavy metal transportation under drought (Chung et al., 2018; Liang et al., 2021). Additionally, it shares the same LD block with GDSL esterase/lipase protein 30 (*OsGELP30*, Os02g0110000) (Chepyshko et al., 2012) and *vacuolar invertase 2* (*VIN2*) (Os02g0106100) genes related to grain size and grain length (Lee et al., 2019). MTA identified for GFe on chromosome 8 at 19.37 Mbp was found proximal to Os08g0403300, reportedly associated with heavy metal transport and detoxification genes (Liang et al., 2021). Barth et al. (2009) reported *HIPP26*, an *Arabidopsis* ortholog of Os08g0403300 which encodes heavy-metal-associated protein in close proximity of this MTA. Out of three significant MTAs on chromosome number 12, MTA at 14.19 Mbp was found closer to *OsMTP1* (Os12g043500) involved in Zn ion binding and transportation (Lyu et al., 2013). Joshi et al. (2024) reported metaQTL MQTL 12.2 within 18,934,992–19,829,588 bp which was closest to MTA identified at 18 Mbp on chromosome 12. Another MTA located at 21.26 Mbp on the same chromosome was found to be associated with genes Os12g0538000 and Os12g0538066 at a distance of 149 and 160 kb, respectively, and both of the genes are found to be associated with metal-binding proteins.

The present study provided valuable genetic information on micronutrient accumulation in rice grains in response to soil Fe variability across multiple sites. To support biofortification efforts through breeding, it is essential to validate the identified candidate genes associated with significant MTAs. There are several methods employed to validate QTLs and candidate genes, such as using early and advanced generation mapping populations, gene cloning and expression analyses, transgenics, gene knockdown, etc (Sharma et al., 2023). In this study, we validated two out of six MTAs (*qGFe12.2* ^BPN-N^ and *qGZn12.1* ^BPN-N^) using an early generation (F_2:3_) population derived from a biparental cross using SSR markers linked to MTAs (present in the same haplotype). Chandel et al. (2015) validated QTLs for grain zinc by using SSR markers and promoted its application in marker-assisted selection for advanced breeding lines in biofortification programs. The identified SSR markers RM1986 and RM170 can be potentially used for the selection of high grain Fe and zinc traits and introgression in elite lines. The gene models for the validated MTAs showed putative candidate gene *OsFUSED*-associated MTA on chromosome 12 that was previously reported to be associated with iron transporter genes like *yellow stripe like 6* (*OsYSL6*) and *Nicotianamine synthase 1* (*OsNAS1*) (Zheng et al., 2010; Kakei et al., 2012). *OsFUSED* is an ortholog to the wheat gene, *TaFUSED*. There were some intermediary proteins in this network whose functions were not defined. Networked to this gene is a metal tolerance protein gene *OsMTP1* located at 14.02 Mbp. *OsFUSED* is associated with iron ion concentration, and the associated *OsMTP1* is involved in heavy metal transport across plasma membranes including Zn, nickel (Ni), and cadmium (Cd) (Yuan et al., 2012). Advancements in understanding the key genes in grain nutrient accumulation will contribute to the development of rice varieties with enhanced Fe and Zn content through breeding, aiding to address global challenges of micronutrient malnutrition more effectively. This study assessed grain Fe and Zn concentrations in brown rice; however, as white (milled) rice is the primary form consumed, analyzing micronutrient retention post-milling would provide more practical relevance. Additionally, the study was conducted under a broader soil and environmental conditions, which could help in using the information on a wider scale.

## 5 Conclusion

In the present study, we studied 174 lines and identified the genomic regions associated with GFe and GZn. This study also revealed the effect of different soil Fe levels on GFe and GZn content, two major micronutrients that are pivotal to biofortification programs. Although high Fe in the soil increased the grain micronutrient status, the assimilation efficiency was found to be significantly reduced under toxic conditions, indicating the effect of stress on grain accumulation of micronutrients. Along with Fe ions, Zn also showed a similar assimilation response under different environments. Soil pH, EC, and Fe content influenced the assimilation micronutrients, defining their role in imparting GSI. The marker–trait associations identified and their corresponding haplotypes can be used for micronutrient enrichment breeding. The identified significant MTAs are potential candidates for larger studies to understand the genetic regulation of Fe response in rice.

## Data Availability

The datasets presented in this study can be found in online repositories. The names of the repository/repositories and accession number(s) can be found in the article/**Supplementary Material**.
