# Supplementary material for: Genome-wide association analysis of grain iron and zinc in rice grown under agroclimatic sites with contrasting soil iron status
Source: Front Plant Sci. 2025 Jul 7;16:1501878. doi: 10.3389/fpls.2025.1501878 (PMC12278063; doi:10.3389/fpls.2025.1501878)
Supplement: Supplementary file 1 [file DataSheet1.pdf]

Supplementary Table S1. Details of the genotypes used to screen for Fe toxicity

S.No	IRG No.	Accession	DNA unique	Variety name	Varietal group	Origin
1	IRG36	IRGC 117551	IRIS 313-9083	Pankhari 203	Basmati/Sadri	India
2	IRG37	IRGC 117327	IRIS 313-10349	CSR-90 IR-2	Indica	India
3	IRG38	IRGC 76296	IRIS 313-9610	Dangar	Aus/boro	India
4	IRG39	IRGC 117346	IRIS 313-10404	K 479-2-3	Indica	India
5	IRG40	IRGC 35154	IRIS 313-8771	Simul Khuri	Aus/boro	India
6	IRG41	IRGC 20709	IRIS 313-9137	ARC 10100	Aus/boro	India
7	IRG42	IRGC 21780	IRIS 313-8554	ARC 11959	Aus/boro	India
8	IRG43	IRGC 22710	IRIS 313-7736	Nona Bokra	Indica	India
9	IRG44	IRGC 26971	IRIS 313-7780	Sona	Indica	India
10	IRG47	IRGC 67707	IRIS 313-8796	Dudh Kadar	Indica	India
11	IRG48	IRGC 52523	IRIS 313-8631	Dudre	Indica	India
12	IRG49	IRGC 49790	IRIS 313-8559	Keeripala Chill Paddy	Indica	India
13	IRG51	IRGC 63113	IRIS 313-8647	Perunel	Indica	India
14	IRG52	IRGC 61667	IRIS 313-8435	UPRH 233	Indica	India
15	IRG53	IRGC 12524	IRIS 313-9609	ARC 10594	Indica	India
16	IRG54	IRGC 12603	IRIS 313-8986	ARC 10754	Indica	India
17	IRG55	IRGC 12631	IRIS 313-9313	ARC 10799	Indica	India
18	IRG56	IRGC 21348	IRIS 313-9176	ARC 11359	Tr. japonica	India
19	IRG57	IRGC 14567	IRIS 313-8999	ARC 11430 B	Intermediate	India
20	IRG58	IRGC 42672	IRIS 313-8946	ARC 11524	Indica	India
21	IRG60	IRGC 40972	IRIS 313-9560	A RC 11857	Indica	India
22	IRG61	IRGC 41068	IRIS 313-9053	ARC 12536	Intermediate	India
23	IRG63	IRGC 41216	IRIS 313-9347	ARC 13778	Indica	India
24	IRG64	IRGC 42256	IRIS 313-9427	ARC 18092	Indica	India
25	IRG65	IRGC 42274	IRIS 313-8982	ARC 18112	Indica	India
26	IRG66	IRGC 12144	IRIS 313-9424	ARC 5840	Indica	India
27	IRG67	IRGC 53715	IRIS 313-9403	Baduie	Indica	India
28	IRG68	IRGC 6179	IRIS 313-8957	BAM 9	Indica	India
29	IRG69	IRGC 67720	IRIS 313-8988	Banikat	Indica	India
30	IRG71	IRGC 45197	IRIS 313-9348	BK 26	Indica	India
31	IRG72	IRGC 45255	IRIS 313-10148	Cauvery	Indica	India
32	IRG73	IRGC 67485	IRIS 313-9484	Chinnor	Indica	India
33	IRG75	IRGC 52184	IRIS 313-8924	Kutta	Indica	India
34	IRG78	IRGC 51932	IRIS 313-9605	NCS 194	Indica	India
35	IRG79	IRGC 62202	IRIS 313-9492	NCS 237	Indica	India
36	IRG80	IRGC 62604	IRIS 313-9400	NCS 964 C	Indica	India
37	IRG81	IRGC 50009	IRIS 313-9351	Para Nellu	Aus/boro	India
38	IRG82	IRGC 61133	IRIS 313-8920	Patala Safed Sunghawado	Indica	India
39	IRG86	IRGC 52261	IRIS 313-9611	Wanga Barugulu	Indica	India
40	IRG87	IRGC 117326	IRIS 313-10348	CSR-89 IR-15	Indica	India
41	IRG88	IRGC 61127	IRIS 313-8757	Nirguni	Indica	India
42	IRG89	IRGC 8948	IRIS 313-8244	Pokkali	Indica	Sri Lanka
43	IRG90	IRGC 39735	IRIS 313-9566	RP 9-4	Indica	India

44	IRG92	IRGC 74782	IRIS 313-8754	Type 50	Indica	India
45	IRG93	IRGC 117357	IRIS 313-10417	UPR 1201-1-20-1	Indica	India
46	IRG95	IRGC 22417	IRIS 313-8603	ARC 12884	Indica	India
47	IRG97	IRGC 50690	IRIS 313-9522	RPW 9-4 (SS 1)	Indica	India
48	IRG98	IRGC 67742	IRIS 313-8731	Nibari	Indica	India
49	IRG105	IRGC 21074	IRIS 313-8386	ARC 10812	Indica	India
50	IRG110	IRGC 10105	IRIS 313-8530	Dhane Burwa	Indica	India
51	IRG112	IRGC 46459	IRIS 313-10150	N 22	Indica	India
52	IRG116	IRGC 52785	IRIS 313-8305	Uraibool	Indica	India
53	IRG120	IRGC 21727	IRIS 313-8585	ARC 11901	Indica	India
54	IRG124	IRGC 6254	IRIS 313-10527	ADT 12	Indica	India
55	IRG126	IRGC 53942	IRIS 313-11493	AR 133	Tr. japonica	India
56	IRG128	IRGC 21082	IRIS 313-10857	ARC 10825	Indica	India
57	IRG132	IRGC 12673	IRIS 313-10676	ARC 10916	Indica	India
58	IRG134	IRGC 21283	IRIS 313-10862	ARC 11281	Tr. japonica	India
59	IRG135	IRGC 21329	IRIS 313-10864	ARC 11338	Basmati/sadri	India
60	IRG137	IRGC 21614	IRIS 313-10869	ARC 11751	Aus/boro	India
61	IRG138	IRGC 21639	IRIS 313-10871	ARC 11777	Aus/boro	India
62	IRG140	IRGC 21837	IRIS 313-10875	ARC 12021	Aus/boro	India
63	IRG141	IRGC 21881	IRIS 313-10876	ARC 12067	Aus/boro	India
64	IRG142	IRGC 21888	IRIS 313-10877	ARC 12079	Aus/boro	India
65	IRG144	IRGC 21929	IRIS 313-10879	ARC 12124	Aus/boro	India
66	IRG149	IRGC 43016	IRIS 313-11302	ARC 14899	Indica	India
67	IRG152	IRGC 41938	IRIS 313-11277	ARC 15129	Aus/boro	India
68	IRG154	IRGC 43174	IRIS 313-11306	ARC 15385	Indica	India
69	IRG156	IRGC 51756	IRIS 313-11443	ARC 18533	Indica	India
70	IRG160	IRGC 20436	IRIS 313-10849	ARC 7001	Aus/boro	India
71	IRG161	IRGC 40914	IRIS 313-11255	ARC 7056	Indica	India
72	IRG163	IRGC 20606	IRIS 313-10852	ARC 7336	Aus/boro	India
73	IRG167	IRGC 52067	IRIS 313-11448	Baramanj	Indica	India
74	IRG168	IRGC 52410	IRIS 313-11454	Bari Sutar	Aus/boro	India
75	IRG169	IRGC 60893	IRIS 313-11596	Bhainsa Mundariya	Indica	India
76	IRG172	IRGC 34861	IRIS 313-11166	Bhut Muri	Aus/boro	India
77	IRG173	IRGC 53889	IRIS 313-11491	Bir Bahadur	Aus/boro	India
78	IRG175	IRGC 74734	IRIS 313-11917	Butnapar	Aus/boro	India
79	IRG178	IRGC 49573	IRIS 313-11409	Cuttack 29	Indica	India
80	IRG179	IRGC 6445	IRIS 313-10534	D 204-1	Aus/boro	India
81	IRG182	IRGC 6688	IRIS 313-10544	Dongrem	Indica	India
82	IRG184	IRGC 19560	IRIS 313-10833	Edakkadan 0-69-27	Indica	India
83	IRG189	IRGC 60960	IRIS 313-11599	Jugray	Indica	India
84	IRG192	IRGC 36778	IRIS 313-11197	K 17-9-1-1	Indica	India
85	IRG193	IRGC 34954	IRIS 313-11168	Kada Chopa	Aus/boro	India
86	IRG195	IRGC 53670	IRIS 313-11489	Kalu T 139	Indica	India
87	IRG197	IRGC 77128	IRIS 313-11963	Karangi	Aus/boro	India
88	IRG198	IRGC 49774	IRIS 313-11414	Karunjeeraga Samba	Indica	India
89	IRG200	IRGC 52168	IRIS 313-11449	Kodia Phul	Aus/boro	India

90	IRG201	IRGC 52456	IRIS 313-11456	Kolamba	Aus/boro	India
91	IRG204	IRGC 74760	IRIS 313-11919	Lakha Kuar	Indica	India
92	IRG205	IRGC 70854	IRIS 313-11828	Lali Gurmatia	Tem. japonica	India
93	IRG207	IRGC 52343	IRIS 313-11452	Local Bhat	Indica	India
94	IRG209	IRGC 35054	IRIS 313-11171	M 142	Aus/boro	India
95	IRG213	IRGC 51854	IRIS 313-11445	NCS 102	Indica	India
96	IRG214	IRGC 62216	IRIS 313-11636	NCS 271 A	Indica	India
97	IRG215	IRGC 62247	IRIS 313-11638	NCS 331	Indica	India
98	IRG217	IRGC 62290	IRIS 313-11640	NCS 458	Indica	India
99	IRG219	IRGC 62373	IRIS 313-11642	NCS 599	Indica	India
100	IRG223	IRGC 62483	IRIS 313-11646	NCS 771 A	Indica	India
101	IRG224	IRGC 62502	IRIS 313-11647	NCS 809 A	Indica	India
102	IRG228	IRGC 19581	IRIS 313-10835	Perunel 0-69-18	Indica	India
103	IRG229	IRGC 28611	IRIS 313-11041	Poongar	Indica	India
104	IRG230	IRGC 53418	IRIS 313-11480	PR 106	Aus/boro	India
105	IRG232	IRGC 39709	IRIS 313-11243	Ratnagiri 45-2	Aus/boro	India
106	IRG234	IRGC 35117	IRIS 313-11173	Sada AUS	Aus/boro	India
107	IRG235	IRGC 52833	IRIS 313-11462	Salsi	Indica	India
108	IRG236	IRGC 46659	IRIS 313-11371	Sathi	Aus/boro	India
109	IRG238	IRGC 53930	IRIS 313-11492	Sirhanti	Indica	India
110	IRG239	IRGC 35157	IRIS 313-11174	SLO 19	Aus/boro	India
111	IRG240	IRGC 35160	IRIS 313-11175	Sona Aus	Aus/boro	India
112	IRG241	IRGC 61059	IRIS 313-11604	Sugarkand	Indica	India
113	IRG243	IRGC 8892	IRIS 313-10608	T 21	Aus/boro	India
114	IRG245	IRGC 61641	IRIS 313-11620	UPRH 197	Indica	India
115	IRG247	IRGC 61503	IRIS 313-11616	UPRH 31	Aus/boro	India
116	IRG248	IRGC 61525	IRIS 313-11618	UPRH 58	Aus/boro	India
117	IRG249	IRGC 52805	IRIS 313-11461	Vaikatharyan	Aus/boro	India
118	IRG250	IRGC 53339	IRIS 313-11479	Vankali	Indica	India
119	IRG251	IRGC 19588	IRIS 313-10836	Vella Peruvazha 0-68-12	Indica	India
120	IRG252	IRGC 46787	IRIS 313-11374	W 398	Aus/boro	India
121	IRG255	IRGC 42557	IRIS 313-11292	ARC 10120	Indica	India
122	IRG256	IRGC 12656	IRIS 313-10674	ARC 10846	Indica	India
123	IRG257	IRGC 21150	IRIS 313-10859	ARC 10939	Indica	India
124	IRG259	IRGC 42651	IRIS 313-11294	ARC 11245	Indica	India
125	IRG260	IRGC 21315	IRIS 313-10863	ARC 11322	Indica	India
126	IRG262	IRGC 21487	IRIS 313-10868	ARC 11571	Basmati/sadri	India
127	IRG263	IRGC 21965	IRIS 313-10880	ARC 12180	Indica	India
128	IRG265	IRGC 22148	IRIS 313-10885	ARC 12559	Indica	India
129	IRG266	IRGC 22288	IRIS 313-10888	ARC 12726	Tr. japonica	India
130	IRG268	IRGC 42720	IRIS 313-11296	ARC 12800	Indica	India
131	IRG271	IRGC 41134	IRIS 313-11259	ARC 13515	Basmati/sadri	India
132	IRG274	IRGC 41288	IRIS 313-11262	ARC 13888	Indica	India
133	IRG275	IRGC 41313	IRIS 313-11263	ARC 13919	Indica	India
134	IRG283	IRGC 41793	IRIS 313-11273	ARC 14860	Indica	India
135	IRG284	IRGC 43009	IRIS 313-11301	ARC 14868	Indica	India

136	IRG285	IRGC 43166	IRIS 313-11305	ARC 15373	Indica	India
137	IRG287	IRGC 43183	IRIS 313-11308	ARC 15403	Indica	India
138	IRG289	IRGC 43269	IRIS 313-11310	ARC 15929	Indica	India
139	IRG293	IRGC 12343	IRIS 313-10668	ARC 7255	Indica	India
140	IRG294	IRGC 45003	IRIS 313-11349	Bachhai kalma	Indica	India
141	IRG301	IRGC 45352	IRIS 313-11354	CAC 75	Indica	India
142	IRG303	IRGC 49524	IRIS 313-11408	Chitrakali	Indica	India
143	IRG305	IRGC 39247	IRIS 313-11240	CR 157-392-4	Indica	India
144	IRG309	IRGC 26850	IRIS 313-10989	Gutti Akkullu	Indica	India
145	IRG310	IRGC 45996	IRIS 313-11361	Kalikalma	Indica	India
146	IRG311	IRGC 46117	IRIS 313-11362	Keya Nunia	Basmati/sadri	India
147	IRG315	IRGC 46236	IRIS 313-11365	Lanjali	Indica	India
148	IRG316	IRGC 46289	IRIS 313-11367	Makaranda Sail	Indica	India
149	IRG317	IRGC 49891	IRIS 313-11418	Matali	Indica	India
150	IRG323	IRGC 10803	IRIS 313-10640	SR 26 B	Indica	India
151	IRG324	IRGC 46698	IRIS 313-11372	Sufal Dhula	Indica	India
152	IRG326	IRGC 46760	IRIS 313-11373	Type 3	Basmati/sadri	India
153	IRG327	IRGC 50192	IRIS 313-11421	Tulasi Bas	Indica	India
154	IRG328	IRGC 60878	IRIS 313-11595	Amakoyali	Indica	India
155	IRG331	IRGC 21463	IRIS 313-10867	ARC 11538	Tr. japonica	India
156	IRG342	IRGC 70840	IRIS 313-11826	Hira Nakhi	Indica	India
157	IRG344	IRGC 19573	IRIS 313-10834	IARI 11387	Tr. japonica	India
158	IRG345	IRGC 53973	IRIS 313-11494	IC 25690	Tr. japonica	India
159	IRG350	IRGC 60982	IRIS 313-11600	Kotodeshi	Aus/boro	India
160	IRG351	IRGC 74318	IRIS 313-11913	Buagkog	Tr. japonica	Philippines
161	IRG354	IRGC 42316	IRIS 313-8458	ARC 18175	Indica	India
162	IRG356	IRGC 42066	IRIS 313-11281	ARC 15505	Indica	India
163	IRG357	IRGC 20370	IRIS 313-10848	ARC 6188	Tr. japonica	India
164	IRG358	IRGC 34967	IRIS 313-11169	Kanai Bashi	Aus/boro	India
165	IRG359	IRGC 20656	IRIS 313-10853	ARC 10028	Tr. japonica	India
166	IRG365	IRGC 24252	IRIS 313-10933	ARC 7281	Basmati/sadri	India
167	IRG366	IRGC 49850	IRIS 313-11417	Lawangai	Intermediate	India
168	IRG372	IRGC 42127	IRIS 313-11283	ARC 15743	Indica	India
169	IRG373	IRGC 51749	IRIS 313-11442	ARC 18502	Indica	India
170	IRG374	IRGC 46612	IRIS 313-11370	Sachi	Indica	India

Supplementary Table S2. Mean for grain micronutrient content for genotypes exposed to multisite evaluation.

	ADT		BAR-N		BAR-S		DEL	
Genotype	GFe	GZn	GFe	GZn	GFe	GZn	GFe	GZn
IRG36	13.81	26.32	18.23	18.66	20.79	36.44	15.2	30.2
IRG37	17.05	28.04	15.81	26.94	19.81	41.11	16	34.2
IRG38	12.94	30.83	16.59	23.29	21.56	38.31	20.5	34.2
IRG39	14.69	32.89	15.9	17.3	25.48	38.56	22.6	25.9
IRG40	16.61	33.7	15.56	15.85	25.48	38.56	20.2	25.1
IRG41	17.83	27.95	16.42	19.29	19.25	36.83	13.6	32
IRG42	15.12	32.63	16.5	23.82	22.89	38.81	21.5	33.8
IRG43	13.73	27.21	14.52	18.76	19.95	35.89	18.2	31.2
IRG44	15.3	27.66	16.59	20.47	22.4	36.14	18.5	28.8
IRG47	13.64	27.59	14.7	17.12	25.97	35.22	21.6	25.9
IRG48	14.08	24.61	17.71	18.8	18.69	36.95	15.2	29.1
IRG49	14.78	25.33	13.06	24.64	19.25	35.47	17.2	24.4
IRG51	13.9	25.15	16.33	22.92	21.07	36.31	19.8	30
IRG52	15.82	26.41	14.09	19.29	18.2	36.44	15.7	31.2
IRG53	12.85	29.3	16.76	23.55	20.58	36.49	19.1	26.1
IRG54	13.81	28.76	14.7	31.88	20.16	36.86	18.5	27.3
IRG55	11.89	27.59	14.44	18.39	21.56	36.29	20.5	32.8
IRG56	16.43	33.07	15.73	19.93	23.73	36.61	16.9	26.7
IRG57	17.48	28.13	15.13	19.29	22.26	36.81	20.5	26.8
IRG58	11.46	28.11	15.13	24.27	20.44	37.28	16.6	36.8
IRG60	12.07	27.3	10.72	24.46	21.91	35.97	19.6	31.1
IRG61	13.9	27.21	17.19	20.2	21.77	35.92	20.1	34
IRG63	12.94	29.21	17.54	19.65	20.23	36.46	16.4	21.7
IRG64	12.94	28.29	15.3	19.85	19.81	35.23	17.1	25.4
IRG65	13.2	24.52	14.87	18.39	19.46	35.55	17.5	28.2
IRG66	14.34	26.4	17.71	30.61	20.86	35.6	10.7	29.6
IRG67	11.54	29.01	15.56	17.48	21.28	35.75	16.1	28.9
IRG68	13.99	28.38	15.64	19.56	21.98	37.75	18.9	38.7
IRG69	12.85	27.59	15.99	18.39	18.62	36.66	19	26.5
IRG71	14.95	27.5	11.94	18.98	18.9	37.42	15.5	25.3
IRG72	17.22	33.27	16.42	31.07	23.38	37.33	19.3	37.7
IRG73	14.25	31.01	16.25	19.02	22.61	36.98	22	34.5
IRG75	12.85	31.82	14.09	22.73	22.26	37.62	17.3	26.5
IRG78	16.78	31.46	13.4	17.93	21.98	36.83	19.1	31.4
IRG79	14.51	32.36	18.74	30.34	22.68	37	19.6	26.4
IRG80	14.34	29.84	16.16	18.39	22.75	35.72	22.2	29.8
IRG81	13.55	25.96	15.38	26.63	24.36	38.19	24.5	29.5
IRG82	13.73	28.56	16.68	19.56	20.75	35.56	15.3	26.8
IRG86	14.25	27.5	16.25	21.83	22.05	37.1	21.2	32.3
IRG87	20.89	31.9	15.56	18.39	26.49	38.91	18.6	34.5
IRG88	18.44	29.12	18.23	20.2	20.16	38.22	15.7	26

IRG89	12.85	32.98	14.26	18.76	25.2	38.29	15.6	31.6
IRG90	17.57	33.63	15.3	17.48	23.66	37.25	19.8	29.4
IRG92	12.42	24.79	15.13	17.48	18.27	38.93	15.8	28.8
IRG93	13.38	28.67	17.88	21.75	19.81	35.94	18	27.9
IRG95	20.76	37.57	15.13	19.08	24.2	37.85	17.8	32.1
IRG97	12.68	32.71	14.35	21.3	23.1	36.39	18.6	30.8
IRG98	13.12	28.31	16.59	19.29	21.98	36.11	21.1	29.2
IRG105	14.43	26.87	15.73	20.2	19.11	36.19	17	26.2
IRG110	12.07	24.43	21.15	23.74	19.95	34.93	18.2	21
IRG112	14.6	28.22	15.47	17.84	25.48	39.87	26.1	47.3
IRG116	18.18	31.91	14.52	18.84	22.33	40.44	12.2	20.5
IRG120	11.98	25.51	14.95	19.74	21.84	36.56	20.9	24.7
IRG124	13.38	26.96	15.38	19.29	19.04	37.13	16.9	30.6
IRG126	14.95	33.07	16.42	18.39	23.94	36.47	23.9	30.3
IRG128	15.47	28.76	16.42	17.48	20.58	36.66	19.1	25.9
IRG132	14.08	31.46	15.56	16.76	21.98	36.44	17.6	25.4
IRG134	16.17	29.66	15.99	17.48	20.58	36.51	33.3	29.7
IRG135	18.09	34.38	15.04	18.39	26.25	37.03	27.2	25
IRG137	14.95	30.47	21.24	24.73	21.21	37.8	18.9	30.6
IRG138	19.67	30.38	19.26	24.18	28.14	39.95	16.3	33
IRG140	14.34	30.51	17.97	20.2	23.52	40.7	17.9	32.1
IRG141	16.74	34.02	19.09	23.37	22.75	38.5	22.3	35.8
IRG142	14.47	29.57	18.14	24.73	26.3	40.42	25.2	46.7
IRG144	21.46	30.54	16.07	19.44	26.09	37.04	22.8	34.7
IRG149	10.63	26.96	18.14	22.01	17.27	35.31	14.5	22.4
IRG152	13.95	28.85	18.05	21.65	18.74	36.77	14.8	30
IRG154	15.96	30.92	15.73	17.84	20.35	36.22	14.8	32.9
IRG156	11.16	31.9	14.01	19.47	27.77	35.85	18.7	27.1
IRG160	21.81	30.72	14.95	19.98	25.04	38.32	24.9	39.3
IRG161	19.54	31.28	15.38	21.34	21.75	36.17	22.5	33
IRG163	13.08	29.66	20.81	24.42	28.96	41.93	22.8	41
IRG167	15.52	28.02	24	26.78	21.33	35.68	16.5	30.8
IRG168	17.79	30.7	16.42	20.25	23.67	38.47	16.4	35.4
IRG169	25.83	37.12	16.16	22.29	29.59	40.87	21.7	23.3
IRG172	14.16	35.07	17.8	19.67	23.57	39.48	20.2	36.2
IRG173	19.19	28.4	20.12	24.24	18.39	35.18	23.8	27.1
IRG175	13.16	28.76	16.76	19.08	18.67	37.7	17.2	40.6
IRG178	23.56	39.56	15.3	18.17	25.18	38.37	19.5	30.7
IRG179	16.83	33.54	18.48	27.77	22.38	38.08	20.4	36.9
IRG182	13.95	36.04	13.66	17.9	27.63	38	19.1	33.4
IRG184	11.77	23.26	14.95	17.27	19.65	36.37	19.5	29.5
IRG189	17.01	28.67	14.18	28.17	25.6	38.12	19.5	29.3
IRG192	15.87	27.68	16.85	19.44	21.61	36.77	22.3	32.4
IRG193	14.47	40.19	14.7	33.3	24.27	39.36	23.1	38.5
IRG195	14.28	34.69	14.95	20.44	23.32	36.39	20.9	26.6
IRG197	14.56	29.3	14.35	17.08	26.09	41.17	27.5	54.6

IRG198	16.83	28.49	15.21	18.62	19.09	37.28	18.7	38.9
IRG200	12.2	30.54	13.83	18.17	24.16	37.84	22.1	33
IRG201	10.63	32.41	14.7	19.98	21.5	37.09	18.3	36.2
IRG204	25.3	31.9	15.64	19.08	27.87	36.43	27.4	27
IRG205	14.91	26.59	16.68	23.56	23.22	40.3	24.6	51.1
IRG207	14.65	38.48	14.26	24.69	23.85	37.73	21.3	39
IRG209	14.82	29.57	14.52	18.44	26.3	39.11	14.8	28.2
IRG213	12.38	18.46	15.38	19.62	28.05	37.98	33.3	28.7
IRG214	13.86	28.49	14.94	19.86	19.65	37.7	19.5	28.7
IRG215	12.99	33.63	13.83	19.53	22.45	37.51	19.3	27.5
IRG217	12.81	27.75	14.78	19.98	24.2	37.85	21.8	37.8
IRG219	14.91	28.29	13.83	19.08	19.58	35.06	15.2	29.8
IRG223	13.78	29.93	16.25	19.98	20.98	36.05	21.4	35.6
IRG224	13.43	29.84	14.78	19.98	19.51	35.08	17.5	33.4
IRG228	13.86	27.59	17.45	19.26	19.93	35.18	19.9	28.3
IRG229	12.2	26.23	22.71	17.9	23.5	36.15	25	33.1
IRG230	14.3	25.78	20.72	17.27	18.39	37.46	17.7	21.9
IRG232	14.21	30.38	17.28	20.89	20.42	35.7	20.6	33.1
IRG234	11.16	29.28	15.81	18.26	24.09	37.82	22	39.5
IRG235	15	30.38	24.09	18.17	19.93	35.6	31.9	34.8
IRG236	9.67	27.3	14.52	17.63	20.91	35.48	11.3	28.3
IRG238	15.96	31.73	17.71	20.93	20.98	36.62	14.2	24.7
IRG239	16.83	30.38	15.47	21.79	24.2	35.38	26	30.7
IRG240	14.21	29.82	14.78	19.08	22.03	35.93	18.7	34.8
IRG241	16.22	34.89	15.47	19.98	23.43	36.72	20.7	26.9
IRG243	13.34	35.5	14.44	19.98	23.85	37.73	21.3	35.5
IRG245	15.43	29.21	13.4	17.72	19.87	35.64	17.7	36.1
IRG247	17.09	41.3	14.26	18.17	24.65	40.35	22.8	43.2
IRG248	15.83	36.76	16.42	24.19	26.03	38.75	24.3	24.5
IRG249	14.25	27.32	14.35	18.94	20.08	38.09	18.9	30
IRG250	14.95	38.21	17.45	19.98	24.77	38.14	22.5	30.4
IRG251	12.25	28.11	17.11	19.98	23.16	37.72	23.3	39.1
IRG252	13.82	27.23	14.01	19.98	18.33	35.35	10.5	28.4
IRG255	13.64	29.84	17.02	22.7	20.36	35.72	16.2	36.6
IRG256	13.99	27.3	19.86	18.62	19.38	35.99	14.6	32.1
IRG257	11.16	30.47	16.07	19.08	20	35.31	15.8	28.1
IRG259	15.96	28.58	14.52	19.08	18.53	35.78	16	25.9
IRG260	16.18	32.98	23.22	26.01	29.67	37.15	29.5	39.8
IRG262	11.9	33.25	17.71	21.79	26.87	39.05	25.5	29.8
IRG263	11.81	28.85	24.26	18.17	19.59	37.05	15.1	27.6
IRG265	12.33	28.94	23.56	19.08	19.66	35.72	16.6	26.4
IRG266	15.65	30.56	12.71	25.28	20.92	38.26	17	35.6
IRG268	12.81	28.58	14.61	19.08	18.53	34.99	14.1	30.1
IRG271	11.29	26.69	16.07	28.72	20.15	35	19	25.3
IRG274	11.46	29.39	16.25	24.92	20.01	36.16	15.7	22.7
IRG275	11.98	26.05	15.21	19.98	19.52	34.98	18.1	34

IRG283	13.43	28.2	14.44	20.62	22.04	36.02	17.5	26.4
IRG284	16.09	31.28	13.15	19.98	21.48	36.68	18.4	25.4
IRG285	15.21	27.5	13.4	18.17	18.54	36.66	14.2	28.2
IRG287	14.34	33.63	17.19	24.69	23.3	37.94	21	22.7
IRG289	15.56	29.03	12.89	14.86	19.73	37.92	15.9	36.8
IRG293	11.63	33.63	16.33	22.38	23.3	36.53	21	31.2
IRG294	10.85	27.23	13.06	17.85	18.33	37.67	13.9	31.2
IRG301	13.9	29.3	14.09	19.21	19.94	35.64	16.2	22.3
IRG303	15.74	37.57	14.01	16.95	28.69	40.34	17.3	27.6
IRG305	17.05	25.68	12.54	16.49	19.52	35.13	15.6	28.6
IRG309	14.43	25.24	17.19	21.48	19.45	35.91	18	27.5
IRG310	15.48	36.22	15.21	19.67	29.74	38.14	18.7	25.8
IRG311	20.63	25.31	14.7	24.38	26.1	38.78	25	40.7
IRG315	15.83	35.43	16.33	19.39	24.7	37.3	19.4	32.9
IRG316	16.44	29.93	17.28	22.38	20.43	36.85	16.9	31.3
IRG317	17.05	29.12	14.09	27.85	19.8	38.16	17.7	29.6
IRG323	17.31	32.63	17.28	21.48	28.62	38.31	28.6	29.7
IRG324	16.26	28.4	10.72	24.19	19.24	35.69	15.2	50.3
IRG326	12.94	23.08	14.09	18.22	17.49	37.03	15.2	24.5
IRG327	11.63	26.22	16.5	19.76	23.51	36.54	21.3	34.2
IRG328	16.26	33.54	13.49	19.67	23.23	37.89	15.1	23.5
IRG331	17.22	40.98	15.56	17.85	25.26	39.42	23.8	37.9
IRG342	17.57	29.88	13.58	23.47	20.39	38.66	15.8	23.8
IRG344	17.14	26.4	16.68	21.48	19.48	35.11	14.5	25.5
IRG345	17.48	32.58	13.15	19.67	22.49	37.18	18.8	29.8
IRG350	17.14	18.82	14.18	21.57	20.18	35.36	15.5	26.4
IRG351	24.65	30.51	15.9	18.67	20.88	36.56	16.5	31.1
IRG354	16.52	33.18	15.38	17.94	22.95	38.78	16.9	24.7
IRG356	13.64	36.85	15.3	21.75	30.23	37.47	27.3	36.6
IRG357	16.96	34.8	15.99	24.1	24.21	38.81	18.7	29.3
IRG358	14.95	33.79	17.28	28.09	23.79	36.64	18.1	26.5
IRG359	18.88	35.23	13.83	15.68	25.68	38.63	20.8	27.9
IRG365	13.29	25.77	15.99	23.65	24.63	36.93	19.3	28
IRG366	13.47	33.79	13.15	16.95	24.28	36.81	18.8	31.3
IRG372	12.68	33.7	17.36	27.82	23.44	36.51	17.7	23.9
IRG373	12.16	27.39	16.76	24.65	23.02	36.36	17.3	29.5
IRG374	14.34	29.84	15.56	17.49	21.76	38.29	17.7	24.2

Supplementary Table S3. Details of the sub-population membership among the mapping panel

Sub-population	Membership-proportion (%)	Members
POP1	10.50%	IRG38, IRG40, IRG168, IRG197, IRG214, IRG236, IRG163, IRG193, IRG238, IRG234, IRG160, IRG239, IRG243, IRG172, IRG358, IRG241, IRG140, IRG175
POP2	5.29%	IRG56, IRG57, IRG134, IRG266, IRG331, IRG344, IRG345, IRG359, IRG357
POP3	34.7	IRG60, IRG72, IRG156, IRG189, IRG259, IRG265, IRG268, IRG350, IRG374, IRG255, IRG309, IRG93, IRG47, IRG86, IRG289, IRG256, IRG120, IRG305, IRG219, IRG68, IRG192, IRG204, IRG90, IRG287, IRG98, IRG73, IRG66, IRG294, IRG303, IRG110, IRG58, IRG39, IRG82, IRG88, IRG223, IRG215, IRG316, IRG64, IRG373, IRG126, IRG293, IRG44, IRG95, IRG354, IRG217, IRG184, IRG173, IRG75, IRG324, IRG79, IRG274, IRG310, IRG71, IRG105, IRG205, IRG230, IRG97, IRG263, IRG251
Admixtures	49.40%	IRG327, IRG63, IRG161, IRG260, IRG48, IRG 210, IRG78, IRG69, IRG356, IRG323, IRG53, IRG317, IRG154, IRG283, IRG228, IRG315, IRG37, IRG178, IRG49, IRG284, IRG167, IRG301, IRG87, IRG372, IRG182, IRG43, IRG52, IRG249, IRG198, IRG224, IRG92, IRG89, IRG124, IRG229, IRG51, IRG81, IRG65, IRG116, IRG275, IRG285, IRG67, IRG351, IRG250, IRG135, IRG61, IRG342, IRG328, IRG252, IRG209, IRG179, IRG112, IRG240235, IRG201, IRG42, IRG248, IRG152, IRG137, IRG54, IRG149, IRG55, IRG257, IRG80, IRG200, IRG128, IRG366, IRG132, IRG247, IRG169, IRG271, IRG365, IRG232, IRG144, IRG326, IRG311, IRG262, IRG36, IRG41, IRG195, IRG207, IRG141, IRG245, IRG138, IRG142

Supplementary Table S4. Candidate gene models identified around the LD block of MTAs associated with phenotypic responses among the association panel used in the study exposed to multisite field evaluation

SNP	SNP locus	Chrom.	Gene Models	Start site	Stop site	Putative Function	References
qGFe12.2	12:21260572	12	Os12g0534000	21230590	21231614	Embryo Development, Shikimate Metabolic Process	Wang et al., 2020
		12	Os12g0533500	21210477	21211501	Short-day Photoperiodism, Seed Germination, Negative Regulation of Gene Expression,	Sun et al., 2017
		12	Os12g0535900	21322246	21323270	Post translational protein modification, Negative flower regulation, Flower development	Han et al, 2014
		12	Os12g0534200	21240404	21241428	Acyl Carrier Activity	Wang et al., 2020
		12	Os12g0535400	21293012	21294036	AB hydrolase	
		12	Os12g0533400	21207193	21208217	Submergence tolerance	Xiong et al, 2012
		12	Os12g0534100	21238294	21239318	Response To Light Response to Cold, tolerance	Baldoni et al, 2022
		12	Os12g0537000	21368785	21369809	DNA Binding, Metal Ion Binding	
		12	Os12g0536000	21324775	21325799	Alkalin phosphatase protein, Magnesium metabolism and carbon fixation	Chen et al.,2022
		12	Os12g0538066	21420866	21421890	DNA Binding, Metal Ion Binding	Wang et al., 2020
		12	Os12g0538000	21410205	21411229	DNA Binding/Metal Ion Binding	Wang et al., 2020
		12	OS12G0533500	21210477	21214801	*VIL1*, grain yield and biomass	Yoon et al., 2021
		12	Os12g0535900	21322246	21324437	*VIP5*, grain yield and biomass	Yoon et al., 2021
		12	Os12g0538600	21469144	21469455	*OSGRX28*	
qGFe2.1	2:488238	2	Os02g0108800	488913	490611	*CYP89*	
		2	Os02g0110200	554579	556320	HPL1, cold tolerance	
		2	Os02g0106900	404250	406672	*OsRLCK58*	
		2	Os02g0111800	637013	640452	PXL1, cold and heat tolerance	

qGFe1.1	1:2693943	1	Os01g0104600	248828	256872	Homolog of Arabidopsis DE-ETIOLATED1 (DET1),	Zang et al., 2016
		1	Os01g0149800	2693231	2695079	*OSMT2A*	Kim and Kang, 2018
		1	Os01g0149200	2665085	2668967	*OSMT2D*	
qGFe8.1	8:19370641	8	Os08g0403300	19244409	19245490	Heavy transport/detoxification domain containing protein	Liang et al., 2021
		8	Os08g0407200	19463943	19469181	Auxin transportation	
qGZn12.1	12:14185774	12	Os12g0433133	14013372	14014396	Similar to PHD-finger family protein, expressed	
		12	Os12g0433200	14015594	14016183	Zinc finger, CCHC-type domain containing protein	
		12	Os12g0433500	14026695	14028895	Pollen Germination, Dopamine Transport, Pollen Development, Regulation of Transport, Lipid Transport	
		12	Os12g0435200	14232905	14452905	Pollen Development	Liu et al., 2017
		12	Os12g0435000	14219584	14439584	Zinc Ion Binding	Lyu et al., 2013
		12	OS12G0433500	14026695	14037353	*OsFUSED*	Yuan et al., 2012
		12	OS12G0435200	14232905	14234854	*CCD4*	
		12	OS12G0434400	14109246	14114260	*LAP1*	

Citations:

Baldoni, E., (2022). Improving drought tolerance: can comparative transcriptomics support strategic rice breeding? Plant Stress, 3, 100058. doi: 10.1016/j. stress.2022.100058.

Chen, C. Q., Tian, X. Y., Li, J., Bai, S., Zhang, Z. Y., Li, Y., et al. (2022). Two central circadian oscillators *OsPRR59* and *OsPRR9*5 modulate magnesium homeostasis and carbon fixation in rice. Mol. Plant, 15(10), 1602-1614. doi: 10.1016/j.molp.2022.09.008

Han, Y., Yang, H., and Jiao, Y. (2014). Regulation of inflorescence architecture by cytokinins. Front. Plant Sci., 5, 669. doi: 10.3389/fpls.2014.00669

Kim, Y. O., and Kang, H. (2018). Comparative expression analysis of genes encoding metallothioneins in response to heavy metals and abiotic stresses in rice (Oryza sativa) and Arabidopsis thaliana. Biosci Biotechnol Biochem. 82(9):1656-1665. doi: 10.1080/09168451.2018.1486177.

Liang, Y., Tabien, R. E., Tarpley, L., Mohammed, A. R., and Septiningsih, E. M. (2021). Transcriptome profiling of two rice genotypes under mild field drought stress during grain filling stage. AoB Plants, 13(4), plab043. doi.: 10.1093/aobpla/plab043

Liu, Y. J., Liu, X., Chen, H., Zheng, P., Wang, W., Wang, L., et al. (2017). A plastid-localized pentatricopeptide repeat protein is required for both pollen development and plant growth in rice. Sci. Rep., 7(1), 11484. doi: 10.1038/s41598-017-10727-x

Lyu, K., Zhu, X., Wang, Q., Chen, Y., and Yang, Z. (2013). Copper/zinc superoxide dismutase from the cladoceran Daphnia magna: Molecular cloning and expression in response to different acute environmental stressors. Environ. Sci. Technol., 47(15), 8887- 8893. doi: 10.1021/es4015212

Sun, L., Di, D., Li, G., Kronzucker, H. J., and Shi, W. (2017). Spatio-temporal dynamics in global rice gene expression (Oryza sativa L.) in response to high ammonium stress. J. Plant Physiol., 212, 94–104. doi: 10.1016/j.jplph.2017.02.006

Wang, W., Ye, J., Ma, Y., Wang, T., Shou, H., and Zheng, L. (2020). OsIRO3 plays an essential role in iron deficiency responses and regulates iron homeostasis in rice. Plants, 9(9), 1095. doi: 10.3390/plants9091095

Xiong, H., Yang, J., and Li, Y. (2012). Identification of submergence-responsive genes in two indica rice genotypes carrying SUB1A-1 but exhibiting differential tolerance. J. Plant Biol., 55, 233-241. doi: 10.1007/s12374-011-0315-9

Yoon, J., Jeong, H. J., Baek, G., Yang, J., Peng, X., Tun, W., et al. (2021). A VIN3-like protein OsVIL1 is involved in grain yield and biomass in rice. Plants, 11(1), 83. doi: 10.3390/plants11010083

Yuan, L., Yang, S., Liu, B., Zhang, M., and Wu, K. (2012). Molecular characterization of a rice metal tolerance protein, OsMTP1. Plant Cell Rep., 31, 67-79. doi: 10.1007/s00299-011-1140-9

Zang, G., Zou, H., Zhang, Y., Xiang, Z., Huang, J., Luo, L., et al. (2016). The De-Etiolated 1 homolog of Arabidopsis modulates the ABA signalling pathway and ABA biosynthesis in rice. Plant Physiol., 171(2), 1259-1276. doi: 10.1104/pp.16.00059

Supplementary Table S5. Mean phenotypic performance of F3 lines for yield related traits under field screening.

$F_{2:3}$ lines	BPN-N		BPN-S		$F_{2:3}$	BPN-N		BPN-S		$F_{2:3}$ lines	BPN-N		BPN-S	
	GFe	GZn	GFe	GZn		GFe	GZn	GFe	GZn		GFe	GZn	GFe	GZn
1	11.1	27.3	13	25.3	29	10.3	24.5	12.6	25.2	55	10.5	23.1	16.5	28
2	10.7	31.8	11.9	28.5	30	10	28.1	13.9	29.6	56	9.5	23.3	12.6	27
3	15.1	31.2	12.3	25.3	31	10	27.2	26.6	26.6	57	10.1	29.4	13.4	28.6
5	12.2	25.8	11.8	27.9	32	10.7	27	13.1	25.5	58	10.5	25.8	14.8	28.9
6	11.2	26.4	13.4	29.6	33	9.3	30	14.4	26.5	59	9.7	23	13.6	25.1
7	11.4	30.1	14.1	28.3	34	9.8	27.6	15.5	26.5	60	9.3	26.2	14.9	30.8
8	13.8	27.7	14.6	26.2	35	11.7	30.5	26	29.1	61	10	26.5	15.8	28.2
9	11	27.6	12.3	25.5	36	9.9	31	12.1	26.1	62	9.5	25.5	18.7	26.2
10	10.9	28.3	10.8	25.1	37	9.2	30.2	17.4	29.1	63	10.5	30.1	17.8	28.2
11	11	27.7	11.6	27	38	10.1	28.2	19.3	32.3	64	10.2	25.4	16.4	30.6
12	10.9	28.5	24.3	31.4	39	9.7	26.5	16.5	26	65	8.9	22.8	19.1	28.5
13	10.9	26.5	13.1	27	40	11.7	29.5	14.7	27	67	9.7	25.6	16.4	26.7
14	10.4	29.7	14	25	41	10.2	31	13.9	24.1	68	10.1	25.9	23.6	30.6
15	10.2	24.7	16.2	27.8	42	10.9	32.6	13.3	26.5	69	10	23.5	16.1	30.4
16	11.3	24.8	14.5	28.4	43	10.2	28.6	14.1	28.4	70	9.2	22.2	27	22.2
17	11.6	33.1	14.7	29	44	9.1	25.7	13.3	27.4	71	12.5	26.8	27.4	28.7
18	9.8	25.9	12.7	24.3	45	12.1	32.3	12.8	25.2	72	9.8	23.8	14.4	29.1
19	9.3	24.3	12.7	22.1	46	10.9	33.8	15.1	30.3	73	11	24.2	18.2	32.3
20	10.4	26.8	12	25.3	47	10.2	30.1	14.3	30.5	74	11	26.5	23.4	34.6
22	9.6	28.8	13.5	26.7	48	9.7	28.7	15.2	28.4	75	10.5	25.8	13.9	31.5
23	11.3	29.4	12.8	25	49	9.4	25.9	12.8	25.9	76	9.9	23.4	15.1	32.1
24	10.4	28.2	13.4	24.1	50	10.4	29.4	12.5	25.7	77	10.4	26.9	15.7	31.8
25	9.8	26.6	15.4	26.3	51	11.6	30.2	14.5	28.8	78	12.1	26.3	13.6	28.4
26	9	22.6	14.3	26.3	52	9.5	27.9	13.6	27.2	79	11.6	26.6	14.6	31.6
27	9.8	26.2	13.3	23.8	53	10.9	27.8	12.8	27.6	80	11.6	26.6	14.7	29.3
28	10.8	24.8	14.4	24.8	54	10.3	24.9	13.4	29.2	81	11.3	25.4	14	28.9

F2:3 lines	BPN-N		BPN-S		$F_{2:3}$ lines	BPN-N		BPN-S		$F_{2:3}$ lines	BPN-N		BPN-S	
	GFe	GZn	GFe	GZn		GFe	GZn	GFe	GZn		GFe	GZn	GFe	GZn
82	10.6	28	14.3	32.7	113	10.9	30.2	20	33.3	194	11.4	33.8	11.8	22.8
83	11.3	29.1	14.9	31.9	115	10.1	27.6	17.1	32.9	195	12.9	29.6	13.4	25.4
84	11.4	26.6	14.1	26.6	116	10.1	28.6	18	28.6	196	12	28.7	12.1	28.1
85	10.3	24.1	13.8	28	118	8.9	28.3	16.6	32.7	199	13.9	32.2	11.8	22.8
86	11	25.1	13.4	29.1	119	11.7	28	18.4	33.6	200	18.7	30.8	11.5	25.1
87	10.1	26.7	14.8	30.9	120	11.7	31.3	14.7	27.3	201	19.5	29.5	13	28.7
88	10	28.5	14.6	28.1	122	15.5	25.3	13	27.5	202	12.2	31.7	13.7	26.8
89	9.9	24.5	14.7	24.3	123	12.4	34	13.5	31.1	203	11.7	29	13.8	24.8
90	11.5	24	15.7	29.3	124	15.5	30.3	15.5	29.7	204	10.8	29.2	14.6	28.2
91	9.9	23.7	13.7	26.3	125	11.4	27.8	13.5	28.4	205	11.5	30	13.8	25.2
93	12.3	31.3	29	35.1	126	17.8	30.1	13.6	23.5	206	9.3	25.2	13.6	29.9
94	11.8	29.7	13.3	28.3	127	15	25	12.9	24.9	207	10.2	34.9	12.3	23.4
95	13.4	25.4	15	26	128	19.1	33.5	12.6	24.7	208	9.7	30.4	19.4	32.5
96	10.8	26.6	13.9	27.3	129	23	29.8	13.1	24.6	209	10.1	27.4	15.4	30.5
97	14.2	27.6	13.9	28.5	130	12.2	34.4	13.6	27.3	211	10.9	33.6	15.3	27.4
98	11.6	26	15.3	28.4	131	12.2	29.9	12.2	27	212	10.9	29.4	15	27.5
99	9.7	23	12.8	26	132	13.2	29	12.8	28.9	213	14.9	31.6	18.4	25.6
100	10.5	24.4	12.4	22.1	133	11.5	28.7	11.5	24.6	214	10.5	30.5	13.2	34.1
101	11.6	27.8	14.7	29.2	134	13.9	30.6	12.6	22.2	215	19.6	29.8	20	24.5
102	11.5	28	17	31.1	135	12.9	28.4	11.7	27.6	216	11	29.7	16.3	41.2
103	9.5	28.2	14.4	28.9	136	10.7	25.37	11.8	24.5	217	12.4	29.2	17.3	27.5
104	10	25.9	14.4	27.2	186	11.6	26.3	12.3	24.2	218	13	25	14.5	34.6
105	10.8	29	15.7	31.6	187	11.4	31.2	12.6	25.6	219	13.4	36	16.3	29
106	9.6	26.4	17.8	31.3	188	13	22.3	13.4	26.3	220	19.7	30.5	11.8	26.5
108	10.4	27.3	17	31.6	190	12.5	34.5	12.8	25.9	221	10.5	30.5	16.4	31.9
112	11.9	32.9	19.5	29.6	193	9.9	30.5	13.4	28.6	222	11.4	28.5	24.6	26.9

F2:3 lines	BPN-N		BPN-S	
	GFe	GZn	GFe	GZn
223	15.8	30	14.7	30.4
224	12.1	26.4	14.4	28.4
225	8.9	25.3	19.6	27.5
226	9.5	26.8	17.4	26.7
227	9.7	32.3	14.5	27.9
228	10.5	31.1	14.7	27.9
230	10.1	29.3	15.7	27
231	10.1	27.2	21.3	29.2
232	9.2	27.5	17.6	30.7
233	11.4	27.3	14.2	27.1
234	12.6	29.9	15.6	27.7
235	10.1	27.2	17.6	27.2
236	10.6	24.6	15.4	24.5
237	11.1	32.1	15.4	26.9
238	11.1	28.3	12.9	26.7
239	9.6	28.7	16	27.6
240	11.7	26.3	14.8	32.3
241	8.9	26.1	14.4	25.2
242	10.1	24.3	15.6	26.5
243	9.5	25.5	14.1	26.1
244	12.8	29.1	15.3	26.7
245	12.1	24.8	20.2	28.6
247	9.5	24.1	16.1	25.8
248	11	24.6	14.8	26.7
249	9.9	23.1	16.1	30
251	8.5	26	18.2	26.7
252	11.3	29.5	17.9	26.7
253	22.5	32.2	15.6	30.3
254	10.7	31.9	18.3	29
255	12.7	28	14.2	29.2
256	13	25.6	15.1	27.8
257	10.9	28.8	15.8	29
261	10.5	29.4	15.6	28.8
264	10.3	28.9	14.9	29.1
281	10	25.8	10.9	27.3
285	10	28.4	10.7	27.7

Supplementary Table S6. Descriptive statistics of $F_{2:3}$ validation population

	Fe	Zn
Mean + S.E	15.2 +0.26	28 +0.23
CV	3.48	3.28
Range	8.1-31.1	22.14-38.9
SD	0.26	0.23
GCV	20.5 (high)	9.49 (low)
PCV	20.76 (high)	10.03 (medium)
h2 (BS)	97.2 (high)	89.55(High)
MSS	9.9**	7.83*

Supplementary Table S7. Markers associated with identified MTAs used for parental polymorphism survey

MTA	Markers	Chrom. No.	Position (Mb)
1:2693943	RM10131	1	2.54
	RM1869	1	2.69
	RM3740	1	2.8
2:488238	RM109	2	0.18
	RGNMS813	2	0.22
	RM12329	2	0.57
8:18677965	RGNMS2825	8	18.04
	RM23032	8	18.6
	HVSSR08-35	8	18.87
	RGNMS2832	8	19.74
	RM22983	8	17.4
	RM23021	8	18.2
	RM23062	8	19.3
12:18503677	RM217	12	17.91
	RGNMS3681	12	18.18
	RGNMS3781	12	18.21
	RM28204	12	18.31
	RM277	12	18.32
	RM28215	12	18.48
12:21260572	RM28326	12	20.24
	HvSSR1236	12	21.17
	RM1986	12	21.28
	RM309	12	21.45
	RM463	12	22.16
12:14185774	RM28032	12	13.64
	RGNMS3746	12	14.4
	RM179	12	14.45
	RM28059	12	14.49
	RGNMS3747	12	14.6
	RGNMS3749	12	15.2
